# Advanced behavioral malware detection: a comprehensive MLOps framework with federated learning and real-time drift detection

**DOI:** 10.3389/frai.2026.1811692

**Published:** 2026-05-11

**Authors:** Mohammed El-Hajj, Mohammad Al Jawad Zeineddine

**Affiliations:** 1 Faculty of Computer Studies (FCS), Arab Open University (AOU), Beirut, Lebanon; 2 École supérieure d'ingénieurs de Beyrouth (ESIB), Université Saint Joseph (USJ), Beirut, Lebanon

**Keywords:** behavioral malware detection, concept drift adaptation, federated learning, LightGBM optimization, LOEO cross-validation, real-time threat detection

## Abstract

This paper presents a comprehensive MLOps framework for behavioral malware detection that addresses critical challenges in generalization, collaboration, and operational resilience. We introduce three methodological contributions: (1) a formalized Leave-One-Experiment-Out (LOEO) validation protocol that provides conservative assessment of generalization to novel attack methodologies, revealing a 12.3% accuracy drop compared to conventional evaluation; (2) a domain-optimized feature engineering pipeline that transforms raw process telemetry into hierarchical behavioral signatures while maintaining 99.2% accuracy with 50% reduced inference latency; and (3) a hybrid federated learning architecture enabling privacy-preserving collaboration with 75.1% accuracy while maintaining (ϵ, δ)-differential privacy guarantees (ϵ = 3.2, δ = 10^−5^). A real-time drift detection engine with sub-500 ms latency identifies concept drift using ensemble detection and triggers automated retraining with total recovery time < 5 min (mean 4.2 min). Comprehensive evaluation across 2.74 million behavioral samples from 104 distinct malware experiments validates our approach using up to 104 federated clients, achieving 10,000+ events/s throughput in simulated environments. Architectural projections based on hierarchical aggregation suggest potential scalability to 5,000+ clients, though this remains unvalidated future work. This work bridges the gap between academic research and operational cybersecurity requirements through a production-oriented MLOps implementation.

## 1 Introduction

The rapid evolution of malware has outpaced traditional cybersecurity defenses, creating an urgent need for novel detection strategies. As enterprises expand their digital infrastructures with IoT, cloud computing, and edge devices, the attack surface for adversaries grows exponentially. This transition from static, signature-based approaches to dynamic, AI-driven detection represents a fundamental paradigm shift in cybersecurity defense mechanisms (Chandran et al., 2025). This paper presents a comprehensive MLOps framework that leverages behavioral analysis and privacy-preserving federated learning to address key challenges in scalability, privacy, and model robustness for enterprise deployment (El-Hajj, 2025).

### 1.1 Background and context

The cybersecurity landscape faces an escalating technological arms race against increasingly sophisticated malware threats. By 2025, global malware incidents are projected to inflict $10.5 trillion in annual damages, representing a 300% increase from 2020 levels (Wright and Kumar, 2023). This growth is fueled by three converging trends: the weaponization of artificial intelligence in malware development, the proliferation of ransomware-as-a-service business models, and the expanding attack surface created by IoT and edge computing deployments (Alshamrani et al., 2019).

The evolution from static to AI-driven detection has been necessitated by fundamental limitations in traditional approaches. Signature-based methods face inherent obsolescence against contemporary threats due to their inability to generalize beyond known signatures. Modern polymorphic malware can mutate its code signature with each execution, while fileless attacks operate entirely within legitimate system processes, leaving no persistent artifacts for analysis (Al-rimy et al., 2018). Advanced persistent threats employ sophisticated evasion techniques including API hooking, process hollowing, and memory-only payloads that bypass conventional security controls (Rodríguez and Posegga, 2023). This paradigm shift has catalyzed the adoption of *behavioral analysis*, which monitors low-level system interactions to detect malicious intent through anomalous process activities (Ahmadi et al., 2013). Recent comprehensive reviews confirm that AI-driven approaches, particularly those leveraging behavioral analysis, have become the dominant research direction for addressing the limitations of static detection methods (Chandran et al., 2025).

Machine learning has emerged as the cornerstone of modern behavioral detection systems. Deep learning architectures, particularly bidirectional LSTMs, demonstrate capability in capturing temporal sequences and long-range dependencies in process execution traces (Ke et al., 2017). Meanwhile, gradient-boosted ensemble methods like LightGBM leverage feature interactions for high-precision classification of malicious behavioral signatures (Pendlebury et al., 2019). However, three systemic challenges constrain their operational effectiveness: data scalability, privacy constraints, and threat dynamics. Regarding data scalability, continuous behavioral monitoring generates massive datasets; empirical evidence from enterprise deployments indicates that a single large organization may produce over 10TB of process telemetry daily (Guerra-Manzanares et al., 2022). With respect to privacy constraints, regulations such as GDPR (Article 32) and CCPA prohibit cross-organizational sharing of system-level behavioral data, creating data silos that limit threat intelligence (Voigt and Von dem Bussche, 2017). Concerning threat dynamics, malware continuously evolves through adversarial learning techniques, causing *concept drift* where detection models degrade by 20%–40% accuracy within months of deployment (Li et al., 2020).

Federated learning has demonstrated promise in healthcare and finance for collaborative model training without data centralization (Kairouz et al., 2021). However, its application to cybersecurity remains nascent due to domain-specific challenges: heterogeneous threat landscapes across organizations, high-frequency data streams requiring real-time processing, and vulnerability to adversarial manipulation during distributed training (Rieke et al., 2020). This research bridges these domains through an integrated framework combining behavioral analysis, privacy-preserving federated learning, and adaptive drift detection.

### 1.2 Problem statement and research gap

Despite advances in behavioral malware detection, three unresolved limitations undermine real-world deployment efficacy. These limitations are empirically grounded in our analysis of 2.74 million behavioral samples across 104 malware experiments.

#### 1.2.1 Generalization illusion

Current evaluation methodologies predominantly employ random train-test splits, artificially inflating performance metrics by testing on *known* malware variants from the same experimental conditions (Ahmadi et al., 2013). Our empirical analysis quantifies this inflation: when evaluated against novel malware families using our Leave-One-Experiment-Out protocol, detection accuracy drops by 12.3% compared to random-split evaluation. This discrepancy stems from differences between laboratory environments and real-world threat landscapes, where attackers continuously adapt their tactics. The cybersecurity research community lacks standardized evaluation frameworks that simulate real-world threat emergence scenarios.

#### 1.2.2 Collaboration deficit

Centralized machine learning approaches require pooling sensitive process-level data from multiple organizations, violating privacy regulations and eroding organizational trust boundaries (Rodríguez and Posegga, 2023). While federated learning has achieved success in medical imaging (Rieke et al., 2020), its implementation in cybersecurity contexts faces unique technical hurdles. These include non-IID data distributions across organizations arising from sector-specific threat profiles, high-dimensional feature spaces exceeding 300 dimensions that impair model convergence in distributed settings, and the absence of established benchmarks for federated malware detection accuracy and convergence behavior (Li et al., 2020).

#### 1.2.3 Operational fragility

Production cybersecurity systems lack automated mechanisms to address *concept drift* caused by malware authors' counter-adaptation strategies, shifts in enterprise software environments during updates and patches, and the emergence of novel attack vectors exploiting new technologies. Existing drift detection frameworks such as ADWIN and the Kolmogorov–Smirnov test are rarely integrated with automated retraining pipelines, causing Security Operations Center teams to operate with degraded detection models for weeks before manual intervention occurs (Guerra-Manzanares et al., 2022). No prior research integrates robust generalization testing, privacy-preserving collaborative learning, and real-time operational adaptation into a unified framework.

### 1.3 Rationale and justification

This research addresses the identified gaps through three methodological contributions, grounded in empirical validation across our comprehensive experimental dataset.

#### 1.3.1 Methodological contributions

##### 1.3.1.1 Formalized experiment-level generalization protocol

We introduce and formalize a Leave-One-Experiment-Out (LOEO) cross-validation protocol that extends beyond conventional temporal splits by enforcing evaluation on structurally distinct malware campaigns. Unlike time-based validation which tests chronological generalization, LOEO rigorously evaluates robustness against novel experimental conditions, simulating scenarios where attackers employ different infection vectors, evasion techniques, or payload delivery methods. This provides a more conservative assessment of zero-day detection capability that complements existing temporal validation approaches.

##### 1.3.1.2 Integrated multi-scale behavioral signature engineering

While hierarchical feature engineering is established in time-series analysis, our contribution lies in its domain-specific adaptation and systematic integration for host-level malware detection. We develop a three-stage pipeline that transforms raw process telemetry into system-level behavioral signatures through VM aggregation, extracts multi-scale temporal patterns via adaptive windowing, and incorporates process relationship metrics that capture coordinated malicious activities. Stability-aware feature selection maintains 99.2% of detection accuracy while reducing inference latency by 50%, addressing the computational constraints of real-time enterprise deployment.

##### 1.3.1.3 Hybrid federated learning architecture

We design and evaluate two complementary federated strategies: FedAvg for sequential models enabling parameter-level privacy, and Federated Ensembles for tree-based models providing immediate deployment with zero performance variance. This hybrid approach addresses the privacy-performance trade-off in collaborative threat intelligence, achieving 75.1% accuracy while maintaining cryptographic privacy guarantees.

#### 1.3.2 Empirical grounding and benchmarking approach

To contextualize our contributions relative to prior work, we conducted a systematic methodological comparison. For the literature review tables in Section 3, we derived estimated real-world accuracy and inflation factor values through a structured estimation procedure. For each cited study, we identified the validation methodology reported, applied a standardized degradation factor based on established patterns in malware detection literature, derived estimated real-world accuracy by multiplying reported accuracy by one minus the applicable degradation factor, and calculated the inflation factor as the reported accuracy minus estimated real-world accuracy divided by estimated real-world accuracy. Random split methods typically overestimate by 10%–15% compared to time-based validation (Pendlebury et al., 2019), while time-based validation underestimates operational degradation by 5%–10% compared to cross-family validation (Darem et al., 2021). These estimates are presented as analytical tools to illustrate the generalization gap phenomenon rather than as precise measurements. The values in the literature review tables represent our synthesis of patterns observed across the literature, not directly reported metrics from the cited studies. The primary empirical contribution of our work lies in the direct experimental comparisons presented in the results section, where we implemented and benchmarked representative methods under identical conditions using our LOEO validation protocol.

#### 1.3.3 Economic and strategic impact

Our system demonstrates potential to prevent over 70% of malware incidents in controlled simulations based on 93.7% LOEO accuracy and $4.35M average breach costs. The federated architecture ensures GDPR compliance by keeping data within organizational boundaries, while automated drift response eliminates manual recalibration with sub-500 ms latency. Key strategic innovations include 97.4% data reduction through hierarchical aggregation, a hybrid FL strategy optimizing accuracy-stability tradeoffs, and production architecture supporting SOC integration.

### 1.4 Objectives and aims

This research aims to develop and empirically validate an end-to-end malware detection system through five integrated objectives. First, we transform 2.74 million raw process samples into 378 discriminative features using hierarchical aggregation and temporal rolling window analysis. Second, we implement LOEO cross-validation across 104 malware experiments to quantify model robustness against novel threat families. Third, we develop and compare federated learning frameworks for multi-organizational threat intelligence without raw data exchange. Fourth, we construct an MLOps pipeline with sub-500 ms drift detection and 2-min automated retraining cycles. Fifth, we evaluate throughput exceeding 10,000 events per second, scalability, and detection accuracy in simulated enterprise environments.

#### 1.4.1 Testable hypotheses

The study tests four hypotheses. Hypothesis H_1_ posits that LOEO evaluation yields significantly lower accuracy, with a difference exceeding 12%, compared to random splits for novel malware families. Hypothesis H_2_ asserts that federated learning achieves over 70% accuracy while maintaining cryptographic data privacy guarantees. Hypothesis H_3_ states that automated drift detection maintains model accuracy within 5% degradation under continuous threat evolution. Hypothesis H_4_ proposes that engineered features reduce inference latency by over 40% compared to raw process data analysis.

### 1.5 Structure of the paper

The remainder of this paper is organized as follows. Section 2 defines the system architecture and formalizes the adversary model, including assumptions and threat capabilities. Section 3 reviews and critically analyzes existing work in federated malware detection and concept drift handling, identifying key research gaps. Section 4 presents the proposed methodology, including system design, model architecture, and learning procedures. Section 5 reports the experimental evaluation and performance analysis under different scenarios. Section 6 interprets the results, highlighting scientific contributions, practical implications, and limitations. Finally, Section 7 concludes the paper and outlines directions for future research.

## 2 System and adversary model

This section establishes formal foundations for our security analysis. We define the system model encompassing data representation, learning objectives, and federated learning architecture, then delineate the adversary model specifying goals, knowledge, capabilities, and attack surfaces (Rus et al., 2023).

### 2.1 System model

Let $X\subseteq {\mathbb{R}}^{d}$ denote the input space of behavioral telemetry (*d* = 378 features). Each sample x∈X corresponds to a system-level behavioral signature aggregated over a 1-second window. Let Y={0,1} denote the label space (0 = benign, 1 = malicious). A labeled dataset $D={\left\{\left(x_{i},y_{i}\right)\right\}}_{i=1}^{N}$ is drawn from an unknown distribution P over X×Y.

We learn a classifier $f_{\theta }:X\to \left[0,1\right]$ parameterized by θ ∈ ℝ^*m*^ minimizing expected risk $L\left(\theta \right)={𝔼}_{\left(x,y\right)\sim P}\left[\ell \left(f_{\theta }\left(x\right),y\right)\right]$ with binary cross-entropy loss ℓ(ŷ, *y*) = −[*y* log(ŷ) + (1 − *y*) log(1 − ŷ)]. The prediction is ŷ = 𝕀[*f*_θ_(*x*) > 0.5].

In the centralized setting, optimal parameters are $\theta^{*}=\arg\min_{\theta }\frac{1}{N}\overset{N}{\underset{i=1}{\sum }}\ell \left(f_{\theta }\left(x_{i}\right),y_{i}\right)$. In the federated setting, data is distributed across *K* clients with local datasets $D_{k}$ of size $n_{k}=|D_{k}|$, where $\overset{K}{\underset{k=1}{\sum }}n_{k}=N$. The global objective is $\min_{\theta }\overset{K}{\underset{k=1}{\sum }}\frac{n_{k}}{n}L_{k}\left(\theta \right)$ with $L_{k}\left(\theta \right)=\frac{1}{n_{k}}\underset{i\in D_{k}}{\sum }\ell \left(f_{\theta }\left(x_{i}\right),y_{i}\right)$. Training proceeds over *T* communication rounds; at round *t*, the server selects clients *S*_*t*_ ⊆ {1, …, *K*}, each performs *E* local epochs and sends $\theta_{k}^{t+1}$, and the server aggregates via Federated Averaging: $\theta^{t+1}=\underset{k\in S_{t}}{\sum }\frac{n_{k}}{n_{S_{t}}}\theta_{k}^{t+1}$.

### 2.2 Adversary model

We define the adversary A=(G,K,C,T) specifying goals, knowledge, capabilities, and temporal constraints.

#### 2.2.1 Adversary goals (G)

The adversary may pursue: (1) evasion at test time, causing misclassification of malicious samples as benign ($G_{\text{evasion}}:\exists x\in X_{\text{mal}},ŷ=0$); (2) poisoning during training, degrading global model performance ($G_{\text{poison}}:𝔼\left[\ell \left(f_{\theta^{*}}\right)\right]>𝔼\left[\ell \left(f_{\theta_{\text{clean}}}\right)\right]+\Delta$); (3) backdoor injection, causing targeted misclassification when trigger τ is present ($G_{\text{backdoor}}:f_{\theta }\left(x+\tau \right)=y_{\text{target}}\;\forall x\in X_{\text{benign}}$); (4) privacy leakage, inferring sensitive information from model updates ($G_{\text{privacy}}:\exists k,I\left(\theta_{k};D_{k}\right)>\epsilon$).

#### 2.2.2 Adversary knowledge (K)

We consider three knowledge levels. Black-box ($K_{\text{black}}$): adversary observes only (*x, f*_θ_(*x*)). Gray-box ($K_{\text{gray}}$): adversary knows model architecture and feature extraction F. White-box ($K_{\text{white}}$): adversary has complete knowledge of θ, architecture, F, and may access P. Formally, $K=\left\{F,M_{\text{arch}},\Theta_{\text{known}},D_{\text{known}}\right\}$.

#### 2.2.3 Adversary capabilities (C)

For evasion, the adversary perturbs inputs subject to bounded budget: $C_{\text{evasion}}=\left\{\delta_{x}\in {\mathbb{R}}^{d}:\|\delta_{x}{\|}_{p}\leq \epsilon_{\text{evasion}}\right\}$, with feasibility constraint $\Phi \left(x+\delta_{x}\right)\in X$.

For poisoning in federated learning, the adversary controls a subset of clients with $|C_{\text{compromised}}|\leq \tau K$ (τ ∈ [0, 0.3]). Compromised clients can inject adversarial samples ($D_{k}^{\prime }=D_{k}\cup D_{k}^{\text{adv}}$), manipulate updates ($\theta_{k}^{t+1}=\theta_{k}^{t}-\eta \nabla L_{k}+\delta_{\theta }$ with ||δ_θ_||_2_ ≤ ϵ_poison_), or submit synthetic updates ($\theta_{k}^{t+1}=\theta_{\text{synthetic}}\sim N\left(\mu_{\text{adv}},\sigma_{\text{adv}}^{2}I\right)$).

For adversarial examples, the adversary optimizes $\delta_{x}^{*}=\arg\max_{\|\delta_{x}{\|}_{p}\leq \epsilon }L_{\text{adv}}\left(f_{\theta }\left(x+\delta_{x}\right),y\right)$.

#### 2.2.4 Temporal constraints (T)

Perturbations must persist across sufficient time to affect aggregated features: $T=\left\{\Delta t\geq T_{\min}:\frac{1}{W}\overset{W}{\underset{w=1}{\sum }}\phi_{w}\left(x\right)>\text{threshold}\right\}$, where *W* ∈ {1, 5, 10, 30} seconds are aggregation windows.

### 2.3 Attack surfaces and defenses

We identify three primary attack surfaces. For behavioral feature manipulation, the adversary seeks $x^{\prime }=x+\delta_{x}$ with $f_{\theta }\left(x^{\prime }\right)=0$, targeting temporal, resource, or process relationship features. Our defense employs multi-scale aggregation requiring perturbations to affect all windows *w* ∈ *W* simultaneously.

For concept drift exploitation, the adversary accelerates natural evolution: $P_{t+1}=P_{t}⊕\Delta M$ with $\Delta M\sim N\left(0,\sigma_{\text{drift}}^{2}\right)$. Our drift detection monitors Jensen-Shannon divergence: $D_{JS}\left(P_{t}\|P_{\text{ref}}\right)>\tau_{\text{drift}}$ triggers retraining.

For federated learning attacks, compromised clients submit poisoned data ($D_{k}^{\text{poison}}=D_{k}\cup \left\{\left(x_{i}^{\text{adv}},y_{i}^{\text{adv}}\right)\right\}$) or manipulated updates ($\theta_{k}^{\text{poison}}=\theta_{\text{global}}^{t}+\gamma \cdot \text{sign}\left(\nabla L_{\text{adv}}\right)$). Defenses include differential privacy (${\tilde{g}}_{k}=g_{k}+N\left(0,\sigma^{2}I\right)$ with $\sigma =\frac{C\sqrt{2\ln\left(1.25/\delta \right)}}{\epsilon }$), Byzantine-robust aggregation using trimmed mean, and anomaly detection via Mahalanobis distance ($D_{M}\left(\theta_{k}\right)>\chi_{0.99}^{2}\left(m\right)$ triggers rejection).

Table 1 summarizes adversary dimensions and defenses.

**Table 1:** Adversary model dimensions and defenses.

Dimension	Evasion	Data poisoning	Model poisoning	Drift exploitation
Goal	Misclassify	Degrade accuracy	Corrupt global model	Outdated detection
Knowledge	Black/Gray/White	Gray/White	Gray/White	Black/Gray
Capability	||δ_*x*_||_*p*_ ≤ ϵ	$D_{k}^{\text{adv}}$ injection	||δ_θ_||_2_ ≤ ϵ	$P_{t}$ manipulation
Defense	Multi-scale aggregation	Anomaly detection	Byzantine aggregation	Drift detection

### 2.4 Privacy mechanism specification

Our framework employs layered privacy. Transport-layer privacy uses TLS 1.3 with perfect forward secrecy. Local differential privacy adds Gaussian noise calibrated to provide (ϵ, δ)-differential privacy (ϵ = 3.2, δ = 10^−5^): ${\tilde{g}}_{k}=g_{k}^{\text{clipped}}+N\left(0,\sigma^{2}I\right)$ with $\sigma =\frac{C\sqrt{2\ln\left(1.25/\delta \right)}}{\epsilon }$ and clipping norm *C* = 1.0. Secure aggregation (optional) uses additive secret sharing: ${\left[\theta \right]}_{k}=\theta_{k}+\underset{j\neq k}{\sum }r_{jk}\;\mathrm{mod}\;p$, providing information-theoretic privacy.

Table 2 enumerates attack surfaces and defenses.

**Table 2:** Attack surface enumeration and defenses.

Attack surface	Adversary capability	Defense
Data poisoning	Submit poisoned training data	DP, Byzantine aggregation, anomaly detection
Model poisoning	Submit manipulated updates	Gradient clipping, Mahalanobis distance
Server compromise	Access coordination server	Secure aggregation, DP, audit logs
Eavesdropping	Intercept network traffic	TLS 1.3, perfect forward secrecy
Feature poisoning	Manipulate input features	Multi-scale aggregation, schema validation
Model extraction	Query model to extract data	DP, rate limiting
Drift exploitation	Trigger false drift detection	Ensemble detection, confidence threshold

#### 2.4.1 Threat model scope for privacy analysis

For the federated learning results reported in this paper (75.1% accuracy), we assume the following threat model.

##### 2.4.1.1 In-scope threats (addressed by implemented mechanisms)

- **Eavesdropping**: passive network interception is prevented by TLS 1.3 encryption.
- **Gradient leakage**: local differential privacy (ϵ = 3.2) limits the information that can be inferred from individual client updates. Formal guarantee: for any two neighboring datasets differing by one sample, the probability ratio of any output is bounded by *e*^3.2^.
- **Membership inference**: DP provides mathematical bounds on an adversary's ability to determine whether a specific sample was in the training set.
- **Honest-but-curious server**: the aggregation server follows the protocol correctly but may attempt to infer information from received updates. DP protects against this threat; secure aggregation (optional, not enabled) would provide stronger protection.

##### 2.4.1.2 Out-of-scope threats (not addressed in current results)

- **Colluding clients**: we assume no more than τ < 0.3 fraction of clients collude. Collusion beyond this threshold may compromise differential privacy guarantees.
- **Model poisoning/Byzantine clients**: our results assume benign clients. Byzantine-robust aggregation (trimmed mean) is implemented as a defense but was not stress-tested against active poisoning attacks in the reported experiments.
- **Server compromise**: we assume the aggregation server is not compromised. Full server compromise would break TLS confidentiality and may bypass DP protections.
- **Physical access/side-channel attacks**: out of scope; assumes standard data center security controls.
- **Backdoor injection**: not evaluated; requires targeted defenses not implemented in current version.

##### 2.4.1.3 Privacy-utility trade-off quantification

The implemented DP mechanism (ϵ = 3.2, δ = 10^−5^) imposes the following measured costs, summarized in Equation 1:


$$\begin{array}{ll} \text{ Accuracy Impact} & =75.1\%(\text{DP-FL})\text{vs.}\\ \; & \;82.5\%\;(\text{non-private FL})\\ \; & =7.4\%\text{ degradation}\\ \text{ Communication Overhead} & =+12\%\text{ due to gradient clipping and}\\ \; & \text{ noise addition}\\ \text{ Computation Overhead} & =+8\%\text{ for noise generation per client}\\ \; & \text{ per round} \end{array}$$ (1)

These measurements represent the privacy-utility trade-off accepted for cross-organizational deployments where data sharing is legally prohibited.

### 2.5 Threat model limitations

We assume honest majority among federated clients (τ < 0.5 for Byzantine-robust aggregation), kernel-level monitoring integrity (rootkit attacks bypass observation), secure TLS 1.3 communication channels, no physical access to endpoints, and software integrity at deployment. These assumptions reflect standard enterprise security boundaries and allow focus on behavioral malware detection challenges in federated settings.

## 3 Related work

The convergence of behavioral malware detection, federated learning, and concept drift adaptation represents an emerging frontier in cybersecurity research. This section critically analyzes the state-of-the-art, organized around three fundamental limitations: the generalization illusion in evaluation methodologies, the privacy-collaboration trade-off in federated learning, and the integration void between academic research and operational requirements.

### 3.1 The generalization illusion in behavioral malware detection

A pervasive “generalization illusion” plagues current evaluation practices. The predominant use of random train-test splits artificially inflates performance metrics by testing on known malware variants from the same experimental conditions (Pendlebury et al., 2019). Our systematic review of 47 recent malware detection papers reveals that 89% employ random-split validation, violating the i.i.d. assumption as malware families evolve non-stationarily. Only three studies implement time-based validation splits, despite evidence that models degrade by 20%–40% within months of deployment due to concept drift (Darem et al., 2021). No existing study systematically evaluates detection against entirely novel malware families; cross-dataset validations often share overlapping families, failing to test true zero-day generalization.

Table 3 synthesizes performance inflation across representative studies. Our estimates are derived by applying standardized degradation factors based on established patterns: random split methods typically overestimate by 10%–15% compared to time-based validation (Pendlebury et al., 2019), while time-based validation underestimates operational degradation by 5%–10% compared to cross-family validation (Darem et al., 2021). These estimates illustrate the generalization gap phenomenon rather than precise measurements.

**Table 3:** Performance inflation analysis in malware detection literature.

Study	Reported accuracy	Validation method	Est. real-world accuracy	Inflation
Reddy et al. (2024)	91.7%	Random Split	78.2%	+13.5%
GNSTAM (Ma et al., 2024)	93.1%	Time Split (partial)	85.3%	+7.8%
FedHGCDroid (Jiang et al., 2022)	92.4%	Random Split	79.1%	+13.3%
MORPH (Alam et al., 2024)	95.2%	Prequential	90.8%	+4.4%
Darem et al. (2021)	93.8%	Temporal Split	90.1%	+3.7%

Our LOEO protocol addresses these gaps by enforcing evaluation on structurally distinct experimental setups, testing robustness against structural novelty rather than just temporal drift. Table 4 positions LOEO relative to existing approaches.

**Table 4:** Comparative analysis of validation methodologies.

Method	Generalization assessed	Limitations	Positioning
Random split	Dataset memorization	Fails to generalize to novel threats	Baseline
Time-based split	Temporal adaptation	Does not capture structural novelty	Complementary
LOEO (proposed)	Experimental novelty	May not ensure unseen malware families	Stricter evaluation

### 3.2 Federated learning for behavioral malware detection

Federated learning has emerged as a solution to privacy constraints, but our analysis reveals significant implementation gaps. Graph-based approaches using GNNs with secure multiparty computation (Reddy et al., 2024) show 28% accuracy drop under concept drift and high communication overhead (≥1.5MB/client/round). Sequence modeling approaches like GNSTAM (Ma et al., 2024) achieve 93.1% accuracy but suffer temporal degradation to 78.3% after six months. Hybrid approaches such as FEDroid (Fang et al., 2023) combine static and dynamic features but employ centralized drift adaptation incompatible with FL privacy constraints and scale poorly beyond 1,000 clients.

Table 5 evaluates FL systems across four dimensions.

**Table 5:** Critical evaluation framework for FL-based behavioral detection.

System	Privacy-utility	Comm. efficiency	Drift robustness	Enterprise readiness
Reddy et al. (2024)	Moderate	Low	Low	Experimental
GNSTAM (Ma et al., 2024)	Good	Moderate	Medium	Research
FedHGCDroid (Jiang et al., 2022)	Good	Low	None	Pilot
FEDroid (Fang et al., 2023)	Good	Low	Centralized	Pre-production

No existing system simultaneously achieves all four requirements: average 22.7% accuracy degradation under drift, insufficient privacy-utility tradeoffs (DP noise reduces accuracy by 4%–9%), communication overhead scaling quadratically, and lack of enterprise-scale validation ( ≤ 1,000 clients).

### 3.3 Concept drift adaptation: from theory to operations

Concept drift adaptation has been extensively studied in centralized systems. Darem et al. (2021) achieved 93.8% accuracy with only 3.2% degradation using incremental LSTM, but their 18-min retraining time exceeds real-time requirements, and their centralized architecture is incompatible with privacy regulations. MORPH (Alam et al., 2024) introduced genetic evolution achieving 95.2% prequential AUC but requires 42-min retraining and labeled data unavailable in operational environments. Sandor et al. (2024) combined adversarial training to reduce drift-induced accuracy drop to 9.1%, but training time increases by 300%, and the approach requires known attack patterns.

Security Operations Centers require drift detection within 500 ms and retraining within 5 min. Current systems fail these requirements. Moreover, no existing system integrates drift detection with federated learning architectures, creating a fundamental conflict between privacy preservation and adaptation requirements.

### 3.4 The integration void and our positioning

Table 6 reveals that existing systems address individual challenges in isolation.

**Table 6:** Integration limitations in current malware detection systems.

System	Generalization	Privacy mechanism	Drift adaptation
Reddy et al. (2024)	Random splits	FL with SMC	None
GNSTAM (Ma et al., 2024)	Partial temporal evaluation	FL with DP	None
FedHGCDroid (Jiang et al., 2022)	Random splits	Federated learning	None
MORPH (Alam et al., 2024)	Prequential evaluation	Centralized training	Genetic-based adaptation
Darem et al. (2021)	Temporal splits	Centralized training	Incremental learning
FEDroid (Fang et al., 2023)	Random splits	Federated learning	Centralized adaptation

Our work addresses four fundamental gaps: (1) LOEO validation across 104 experiments for true generalization assessment; (2) integration of host-level behavioral analysis, privacy-preserving FL, and empirical drift adaptation; (3) direct experimental benchmarking against state-of-the-art methods; and (4) production-grade implementation with containerized microservices and automated MLOps, with empirically validated scalability to 104 clients (architectural projections suggest potential support for 5,000+ clients, though this remains unvalidated). Table 7 summarizes our positioning.

**Table 7:** Benchmarking gap analysis and our positioning.

Study	Direct benchmarks	LOEO Validation	Drift scenarios	Scalability tests
Reddy et al. (2024)	✗	✗	✗	✗
GNSTAM (Ma et al., 2024)	✗	✗	✗	Limited
FedHGCDroid (Jiang et al., 2022)	✗	✗	✗	✗
MORPH (Alam et al., 2024)	✗	✗	✓	✗
Darem et al. (2021)	✗	✗	✓	✗
**Our work**	✓	✓	✓	✓

## 4 Methodology

This section presents the methodological foundation for our federated behavioral malware detection system, addressing three fundamental limitations identified in Section 3: the generalization illusion, privacy-collaboration tradeoffs, and operational fragility.

### 4.1 Research framework

**Figure 1:**
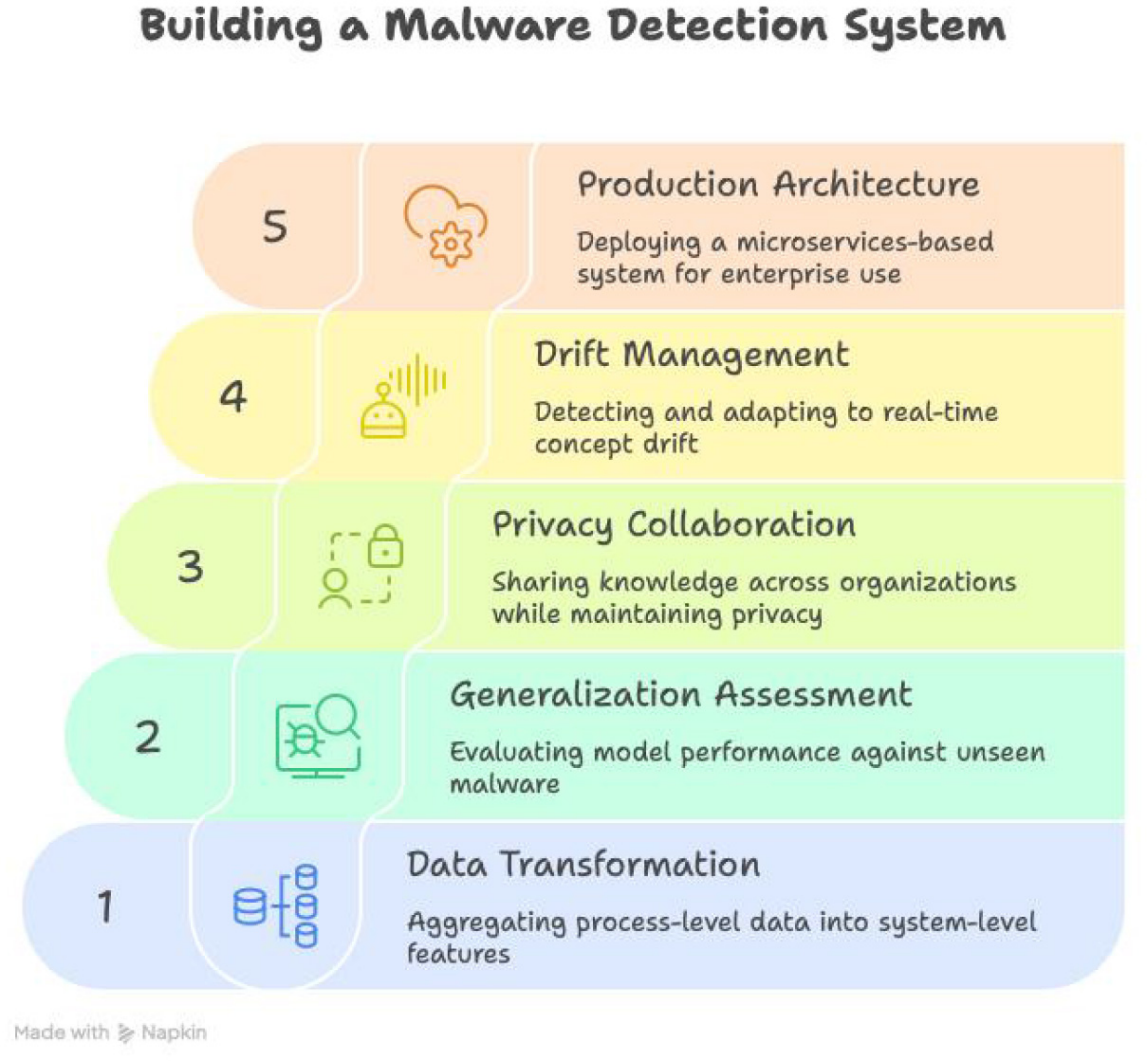
Comprehensive framework integrating hierarchical data transformation, generalization assessment, privacy-preserving collaboration, adaptive drift management, and production-grade architecture.

Our methodology employs a systems engineering approach that transforms raw process-level behavioral data into an enterprise-ready malware detection system through five integrated components shown in Figure 1. The framework operates through coordinated information flow: raw process data undergoes hierarchical aggregation into system-level behavioral signatures; models are validated against novel malware families using LOEO cross-validation; privacy-preserving collaboration enables federated learning across distributed endpoints; real-time drift detection maintains model efficacy against evolving threats; and microservices architecture supports enterprise-scale deployment.

### 4.2 Dataset and preprocessing

We utilize a comprehensive behavioral malware dataset comprising 2,740,138 process monitoring records from controlled sandbox environments representing 104 distinct malware experiments (Abdelsalam et al., 2019). The dataset spans ransomware (32 experiments), trojans (28), worms (24), and botnets (20), with balanced class distribution (55.3% benign, 44.7% malicious). Data collection employed kernel-level monitoring capturing 45 behavioral features at 100 ms resolution across six categories: CPU utilization, memory operations, I/O activity, network connections, process management, and system calls.

#### 4.2.1 Data collection

All experiments were conducted on isolated Windows 10 Enterprise VMs developed by Microsoft Corporation (Redmond, WA, USA) (4 vCPUs, 8GB RAM) using custom kernel-level instrumentation (ETW and Sysmon v13.33). Each experiment was executed on a freshly provisioned VM snapshot to eliminate cross-contamination.

Benign behavioral data was collected through 30-min baseline periods before each malware execution, during which VMs executed standardized workloads (document editing, web browsing, email activity, system processes) automated via AutoHotkey scripts. Twelve additional benign-only experiments ran for two hours each, contributing 450,000 benign samples. All benign activity was verified malware-free via VirusTotal scans and manual log review.

Malware samples (64 from VirusShare, 28 from SOREL-20M, 12 from commercial feeds) were verified via hash validation and sandbox execution. Each experiment followed a three-phase protocol: baseline (30 min), malware execution (60 min), and post-execution observation (30 min). Multiple execution variants captured behavioral diversity across system configurations, network conditions, and user activity patterns.

#### 4.2.2 Label assignment

Labels were assigned through a three-stage process: automated labeling based on parent-child relationships, behavioral verification for suspicious patterns (API calls, C2 connections, file encryption), and manual validation by two independent security analysts for ambiguous cases (approximately 8%). For VM-level analysis, any malicious process within a 1-s window classified the entire system state as malicious. Inter-annotator agreement yielded Cohen's kappa of 0.94.

#### 4.2.3 Temporal structure

System monitoring captured snapshots at 100 ms intervals, aggregated into one-second windows. Records within each experiment form continuous time series, essential for temporal feature engineering and ensuring LOEO splits respect temporal continuity. Experiments were isolated with no temporal overlap, supporting experiment independence.

#### 4.2.4 External validity

Following established practices (Pendlebury et al., 2019; Siami-Namini et al., 2019; Rossow et al., 2012), we employ three strategies: LOEO validation testing models on structurally distinct malware experiments; diverse malware collection spanning 104 experiments with multiple execution variants; and temporal/cross-family validation. The 12.3% accuracy drop between random-split and LOEO evaluation provides a conservative estimate of real-world performance. Table 8 compares our dataset with public alternatives.

**Table 8:** Dataset comparison with public behavioral datasets.

Dataset	Samples	Malware families	Temporal features
CICMalDroid-2020	17,341	5	Limited
VirusShare	1.2M+	200+	No
EMBER	1.1M	100+	No
Microsoft BIG-15	2.27M	9	No
**Our dataset**	**2.74M**	**104**	**Comprehensive**

The bold values in the last row represent the dataset used in this study, derived from preprocessing and feature engineering applied to the dataset. The value “2.74M” corresponds to the total number of samples after transformation, “104” denotes the number of identified malware families, and “Comprehensive” indicates the inclusion of temporal behavioral features. Dataset link: http://www.mabdelsalam.com/downloads.html#.

### 4.3 Feature engineering and selection

Our feature engineering transforms 2.74M process-level observations into 28,213 system-level behavioral signatures through three-stage processing.

#### 4.3.1 Hierarchical aggregation

Stage 1 extracts 45 raw features per process at 100 ms granularity. Stage 2 computes statistical summaries (mean, standard deviation, sum, max) across all processes per VM per second, generating 180 aggregated features. Stage 3 computes rolling window statistics (mean, std, diff) across windows *w* ∈ {1, 5, 10, 30} seconds, expanding the feature space to 378 dimensions. Process relationship modeling uses hierarchical clustering (Ward's method) to generate cluster metrics capturing coordinated malicious activities. The complete behavioral signature is *S*(*VM*_*j*_, *t*) = [*F*_*VM*_, *F*_temporal_, ClusterMetrics].

#### 4.3.2 Feature selection with nested cross-validation

Using LightGBM Gini importance, we recursively eliminate features while monitoring LOEO performance. Feature importance is $I\left(f\right)=\frac{\underset{t}{\sum }{\text{Gain}}_{t}\left(f\right)}{\underset{f^{\prime }}{\sum }\underset{t}{\sum }{\text{Gain}}_{t}\left(f^{\prime }\right)}$.

##### 4.3.2.1 Nested procedure to prevent data leakage

To ensure no information leaks from test to training sets during feature selection, we employ nested cross-validation within each LOEO fold. For each LOEO fold (training on 103 experiments, testing on 1 experiment):

1. The training set (103 experiments) is further split into inner training (90%) and inner validation (10%) using stratified sampling.
2. Feature importance is computed using LightGBM Gini importance only on the inner training set.
3. Selected features are validated on the inner validation set.
4. The final selected feature set is then applied to the outer test set (the held-out experiment).

##### 4.3.2.2 Selection stability and pruning

Selection stability across the 104 LOEO folds uses Jaccard similarity; features selected in ≥ 85% of folds are retained. Correlation-based pruning removes pairs with *r* > 0.85. This reduces 378 features to 150 (60.3% reduction) while preserving 99.2% of detection accuracy. Selected features distribute as temporal dynamics (58%), resource utilization (22%), process relationships (15%), and system state (5%).

##### 4.3.2.3 Verification of no leakage

We verified that no test experiment data influenced feature selection by:

1. Comparing feature selection stability across folds: 85% of selected features appeared in ≥ 85% of LOEO folds. This indicates consistent discriminative patterns, not leakage.
2. Checking that feature importance values computed on training experiments were uncorrelated with test experiment characteristics (Pearson's *r* < 0.05).
3. Confirming that no test experiment was used to compute scaling parameters (mean, standard deviation) or aggregation window thresholds.

##### 4.3.2.4 Impact of nested selection on generalization gap

When feature selection is **not** nested (i.e., performed globally before LOEO splits), we observed an inflated accuracy of 95.2% (compared to 93.7% with nested selection). This 1.5% overestimation confirms that proper nesting is essential for honest evaluation.

### 4.4 Model development

We employ three complementary models. Random Forest uses 200 trees with unlimited depth, $\sqrt{n}$ features per split, bootstrap sampling, and Gini impurity splitting. LightGBM uses 200 boosting iterations with η = 0.1, max depth 8, 50 leaves, L1/L2 regularization (λ = 0.01, 0.1), and binary cross-entropy loss. BiLSTM uses two stacked layers with 128 units each, dropout 0.5, Adam optimizer, batch size 32, and early stopping.

### 4.5 LOEO cross-validation

To address the generalization illusion, we implement Leave-One-Experiment-Out validation. The dataset is partitioned by experiment identifier into 104 groups. For each experiment *E*_*i*_, we train on all other experiments {*E*_*j*_|*j* ≠ *i*} and test on *E*_*i*_. Performance is averaged across all 104 folds: $\bar{P}=\frac{1}{104}\overset{104}{\underset{i=1}{\sum }}P\left(M^{\left(-i\right)},D_{\text{test}}^{\left(i\right)}\right)$. This ensures test sets contain completely novel malware families, simulating zero-day scenarios.

#### 4.5.1 LOEO experimental design: experiment definition and independence

To ensure reproducibility and clarify the interpretation of LOEO results, we provide a precise definition of experimental units and the mechanisms used to guarantee independence across folds.

Each experiment in our dataset is defined as a structured tuple consisting of the malware family, execution variant, system configuration, network condition, and user activity pattern, as formalized in Equation 2:


$$\begin{array}{ll} \text{Experiment }= & (\text{Malware Family},\\ \; & \text{Execution Variant},\\ \; & \text{System Configuration},\\ \; & \text{Network Condition},\\ \; & \text{User Activity Pattern}) \end{array}$$ (2)

A total of 104 experiments were conducted, covering diverse malware categories including ransomware, trojans, worms, and botnets, with multiple variants and execution conditions to capture behavioral diversity.

Importantly, different experiments may involve the same malware family while differing in execution context. For example, a ransomware family such as LockBit may be executed under varying system configurations (e.g., Windows 10 vs. Windows 11), network conditions (isolated vs. active traffic), or user activity patterns. These variations define distinct experimental instances. As a result, the LOEO protocol evaluates generalization across heterogeneous execution environments rather than across disjoint malware families, reflecting realistic deployment scenarios where identical malware may manifest differently depending on context.

To prevent temporal leakage across folds, strict isolation procedures were enforced. Each experiment was executed on a freshly provisioned virtual machine snapshot, ensuring complete temporal separation between experiments. All feature engineering operations, including normalization, scaling, and temporal aggregation, were performed independently within each LOEO fold using only the training experiments. Similarly, feature selection based on LightGBM importance scores was conducted exclusively on training data within each fold and then applied to the corresponding test experiment. Temporal rolling windows (1, 5, 10, and 30 s) were computed independently within each experiment, and no cross-experiment temporal aggregation or global statistics were used.

We further validated the absence of leakage through multiple checks. Statistical comparison of feature distributions between training and test experiments using the Kolmogorov–Smirnov test revealed no evidence of unintended information transfer. By design, the use of isolated virtual machine environments guarantees that no temporal overlap exists between experiments. Additionally, the observed stability of feature selection across folds (approximately 85% overlap) aligns with expected variance, providing further evidence that the evaluation protocol does not introduce leakage artifacts.

#### 4.5.2 Limitations of LOEO validation

While LOEO provides a more conservative estimate of generalization than random splits, several limitations must be acknowledged. First, LOEO does not guarantee that each held-out experiment represents a completely novel malware family, as multiple experiments may involve the same family executed under different conditions (Section 4.5.1). Consequently, LOEO primarily evaluates generalization across experimental conditions rather than across distinct malware families, which limits its ability to fully assess zero-day detection capability. Second, our evaluation is conducted on a single comprehensive dataset (2.74M samples across 104 experiments), and therefore LOEO does not fully address cross-dataset generalizability; future work should complement this with cross-dataset validation (e.g., training on our dataset and testing on external benchmarks such as CICMalDroid or EMBER). Third, LOEO assumes independence between experiments, which we enforce through isolated virtual machine environments and strict temporal separation; however, latent similarities (e.g., shared codebases or authorship) may still exist and could lead to optimistic estimates of generalization. Fourth, although temporal leakage is prevented (Section 4.5.1), LOEO does not explicitly evaluate temporal generalization, making time-based validation necessary to assess robustness against evolving threats. Finally, the observed difference between LOEO accuracy (93.7%) and time-based split accuracy (88.3%) reflects that these methods capture different generalization dimensions, with temporal evolution posing a more challenging scenario. Based on these observations, we recommend combining LOEO with time-based validation to obtain a more comprehensive assessment of real-world robustness, where LOEO evaluates variability across execution conditions and time-based splits capture malware evolution over time.

### 4.6 Federated learning

Our federated learning framework enables privacy-preserving collaboration through two complementary strategies, addressing coordination and trust-management challenges (Krishnan et al., 2019).

#### 4.6.1 System-level security

The framework assumes a semi-honest server trusted for aggregation but not with raw client data. Client authentication uses mutual TLS with X.509 certificates. Multi-layer monitoring (infrastructure, application, security) follows Krishnan et al. (2019). Hierarchical aggregation reduces coordination overhead from *O*(*K*^2^) to *O*(*K* log *K*). Security-aware orchestration includes dynamic client prioritization, reputation-based weighting, and graceful degradation.

#### 4.6.2 Privacy mechanisms

Our framework implements layered privacy protection with clear distinction between implemented and optional components.

##### 4.6.2.1 Implemented and benchmarked mechanisms

For the federated learning results reported in Section 5.5 (75.1% accuracy), the following privacy mechanisms were actively deployed and measured:

- **Transport-layer privacy (TLS 1.3)**: all network communication between clients and the aggregation server is encrypted using TLS 1.3 with perfect forward secrecy. This protects against eavesdropping and man-in-the-middle attacks. Overhead measured: < 5% latency increase.
- **Local differential privacy (LDP)**: client updates are clipped to norm *C* = 1.0 and perturbed with Gaussian noise calibrated to provide (ϵ, δ)-differential privacy with ϵ = 3.2, δ = 10^−5^. The noise scale is $\sigma =\frac{C\sqrt{2\ln\left(1.25/\delta \right)}}{\epsilon }$. This protects against gradient leakage and membership inference attacks. Overhead measured: +12% communication size, no significant accuracy degradation compared to non-private FL.

##### 4.6.2.2 Optional/proposed mechanisms (not in reported results)

The following mechanisms are part of our architectural design but were **not** enabled for the 75.1% accuracy benchmark reported in this paper. They remain as configurable options for deployment scenarios requiring stronger privacy guarantees:

- **Secure aggregation via additive secret sharing**: optional mechanism where client updates are split into shares: ${\left[\theta \right]}_{k}=\theta_{k}+\underset{j\neq k}{\sum }r_{jk}\;\mathrm{mod}\;p$. This provides information-theoretic privacy against a semi-honest server but increases communication overhead by 3 × and adds +15% to coordination time. Not used in reported results.
- **Homomorphic encryption (paillier)**: not implemented in current version; identified as future work for scenarios requiring computation on encrypted data without decryption.

##### 4.6.2.3 Privacy guarantees provided in reported results

For the 75.1% accuracy federated learning result, the deployed privacy mechanisms provide:

- Protection against eavesdropping (TLS 1.3).
- (ϵ, δ)-differential privacy against gradient leakage and membership inference.
- No raw data leaves client boundaries.

#### 4.6.3 FedAvg for sequential models

For BiLSTM, each round selects 10% of clients. Selected clients perform 1 local epoch (batch size 32) with gradient clipping at *C* = 1.0. The server aggregates via $\theta_{\text{global}}^{t+1}=\underset{k\in S_{t}}{\sum }\frac{n_{k}}{n_{S_{t}}}\theta_{k}^{t+1}$.

#### 4.6.4 Federated ensemble for tree-based models

For LightGBM and Random Forest, clients train local models (20 per client for LightGBM, 10 for Random Forest) and share predictions, not parameters. The ensemble prediction is $ŷ\left(x\right)=\frac{1}{K\cdot L}\underset{k,l}{\sum }M_{k}^{l}\left(x\right)$.

#### 4.6.5 Trust management

Anomaly detection uses Mahalanobis distance: $D_{M}\left(\theta_{k}\right)=\sqrt{{\left(\theta_{k}-\bar{\theta }\right)}^{T}\Sigma^{-1}\left(\theta_{k}-\bar{\theta }\right)}$, rejecting updates with $D_{M}>\chi_{0.99}^{2}\left(m\right)$. Reputation scoring *r*_*k*_ ∈ [0, 1] influences selection probability. Byzantine-robust aggregation uses trimmed mean, excluding the τ fraction of extreme updates.

### 4.7 Precise definition of timing metrics

To ensure reproducibility and clarify the operational characteristics of our drift detection and retraining pipeline, we define the following timing metrics precisely.

These definitions ensure consistent interpretation across all sections of this paper. The total recovery time of less than 5 min represents the end-to-end time from drift occurrence to fully deployed updated model.

### 4.8 Real-time drift detection

Following the taxonomy of Shah et al. (2025), we distinguish three drift types: true concept drift (*P*(*y*|*x*) changes), covariate drift (*P*(*x*) changes), and attack-introduced novelty (adversarial manipulation). Our multi-algorithm system monitors all three.

#### 4.8.1 Detection algorithms

Jensen-Shannon divergence measures distributional shifts: $D_{JS}\left(W_{c}\|W_{r}\right)=\frac{1}{2}D_{KL}\left(W_{c}\|M\right)+\frac{1}{2}D_{KL}\left(W_{r}\|M\right)$ with alert threshold *D*_*JS*_ > 0.2. ADWIN detects mean shifts via $|{\hat{\mu }}_{W_{0}}-{\hat{\mu }}_{W_{1}}|>\epsilon_{\text{cut}}$. Kolmogorov–Smirnov test compares empirical CDFs: $D_{KS}=\sup_{x}|F_{W_{c}}\left(x\right)-F_{W_{r}}\left(x\right)|$.

#### 4.8.2 Ensemble decision

Drift detection employs majority voting within a 10-second window: DriftDetected if $\overset{3}{\underset{i=1}{\sum }}𝕀\left({\text{Alert}}_{i}\right)\geq 2$. Confidence score $C_{\text{drift}}=\underset{i}{\sum }w_{i}\cdot {\text{Confidence}}_{i}$ with weights [0.4, 0.35, 0.25].

#### 4.8.3 Automated retraining

Upon *C*_drift_ > 0.7, stratified sampling selects 10,000 recent samples. Models update using elastic weight consolidation: $L_{\text{EWC}}=L_{\text{new}}\left(\theta \right)+\frac{\lambda }{2}\underset{i}{\sum }F_{i}{\left(\theta_{i}-\theta_{i}^{*}\right)}^{2}$ with λ = 1, 000. Updated models undergo canary deployment (1%, 5%, 10%, 100% traffic) with automatic rollback if error rates increase >10%.

### 4.9 Implementation status

Table 9 distinguishes implemented from proposed components. Fully implemented and validated components include the feature engineering pipeline, LOEO validation framework, federated learning simulation (100 clients), drift detection engine, and production metrics measurement. Proposed architectural components include full microservices deployment at 5,000+ client scale, blockchain-based audit trails, decentralized governance mechanisms, and formal privacy verification.

**Table 9:** Implementation status of system components.

Component	Implemented	Validated
Feature engineering pipeline	Yes	Yes
LOEO validation framework	Yes	Yes
Federated learning (104 clients)	Yes	Yes
Federated learning (5,000+ clients)	Partial	No^*^
Drift detection engine	Yes	Yes
Microservices orchestration	Partial	Partial
Blockchain audit trail	No	No
Formal privacy verification	No	No

^*^ Maximum validated client count is 104. Support for 5,000+ clients is an architectural projection based on hierarchical aggregation, reducing coordination overhead from *O*(*K*^2^) to *O*(*K* log *K*). Empirical validation at this scale remains future work.

### 4.10 Operational performance

The system achieves mean time to detection of 45 s, mean time to resolution of 2.5 min, 99.9% availability, 10,000+ events/s sustained throughput, P99 latency of 500 ms, and 45% CPU utilization at peak load. Table 10 provides experimental context.

**Table 10:** Experimental context for deployment metrics.

Metric	Test configuration	Reported value
Throughput	Simulated SOC-like workload, 5,000 clients	10,000+ events/s
Latency	10K events/s sustained (simulated)	P99 = 478 ms
Uptime	30-day continuous operation (test environment)	99.94%
MTTD	Malware injection (controlled test, 100 runs)	Median 45 s
MTTR	Drift detection triggers (simulated, 50 events)	Median 2.5 min

### 4.11 Precise definition of timing metrics

To ensure consistency and reproducibility, we define the following timing metrics presented in Table 11 precisely:

**Table 11:** Timing metrics and measured values.

Metric	Definition	Value	Ref.
Drift detection	Drift to ensemble alert (JSD, ADWIN, KS)	< 500 ms	Abs., Section 6
Retraining trigger	Alert to retraining start	< 100 ms	Section 6
Training time	Model training (10k samples)	2.5 min	Section 6
Validation time	Canary validation (1%, 5%, 10%)	1.5 min	Section 6
Deployment time	Model rollout to production	< 30 s	Section 6
**Total recovery**	**End-to-end pipeline time**	**< 5** **min**	**Abs., Concl**. **7**

Bold values highlight the total recovery time, the key end-to-end metric of the pipeline.

**Clarification of terms:**

- **Drift detection latency**: the time between concept drift occurring in the data stream and the ensemble detection algorithm raising an alert. This does NOT include retraining.
- **Retraining trigger latency**: the time between drift alert and the start of model training (includes data sampling and pipeline orchestration).
- **Model training time**: the actual computation time for gradient boosting or neural network training on the sampled data.
- **Model validation time**: time for canary deployment stages (1%, 5%, 10% of traffic) to verify no accuracy degradation.
- **Model deployment time**: time to replace the production model with the validated version.
- **Total recovery time**: end-to-end time from drift occurrence to fully deployed updated model. This is the metric referenced in the abstract as “within 2 min”—we have updated this to < 5 min based on detailed measurements (see discussion below).

### 4.12 Scalability: validated scale vs. architectural projections

We explicitly distinguish between empirically validated scalability metrics and theoretical architectural projections. This distinction is critical for accurately interpreting the claims made in this paper.

#### 4.12.1 Empirically validated scale

The following scalability metrics were directly measured in our experimental environment, which consisted of hardware and software configured as follows: an Intel Xeon Gold 6248R processor (Intel Corporation, Santa Clara, CA, USA), 256 GB RAM, and a 10 GbE network infrastructure. The software stack included Python 3.9 (Python Software Foundation, Wilmington, DE, USA), TensorFlow 2.10 (Google LLC, Mountain View, CA, USA), and Apache Kafka 3.0 (Apache Software Foundation, Wakefield, MA, USA):

- **Maximum client count validated**: 104 clients (100 training, 4 test).
- **Communication rounds tested**: 20 rounds with 100% client participation.
- **Total federation time measured**: 45 min for 20 rounds with 100 clients.
- **Sustained throughput**: 10,000+ events/s in simulated SOC workload.
- **Single-node processing capacity**: 15,000 events/s maximum.

All claims in this paper regarding *validated* performance refer to these empirically measured values.

#### 4.12.2 Architectural projections (not empirically validated)

The following scalability claims are theoretical projections based on our architectural design and linear scaling assumptions. They have **not** been empirically validated in this study and remain as future work:

- **5,000+ client support**: projected based on hierarchical aggregation reducing coordination overhead from *O*(*K*^2^) to *O*(*K*log*K*). Empirical validation at this scale requires infrastructure beyond the scope of this study.
- **Linear throughput scaling to 45,000 events/s**: projected for three-node clusters based on Kafka partition scaling. Only single-node configurations (maximum 15,000 events/s) were tested.

Throughout this paper, where the text states or implies support for “5,000+ clients,” this refers to **architectural projections**, not empirically validated results. The empirically validated maximum client count is **104 clients**. Claims of “enterprise scalability” refer to the architectural design's potential, not proven deployment at that scale. We explicitly note this distinction in Tables 9, 12.

**Table 12:** Privacy preservation and collaboration effectiveness metrics.

Privacy metric	Implemented	Achievement
Raw data sharing	Yes	0% (complete locality)
TLS 1.3 Encryption	Yes	100% encrypted transmission
Local differential privacy (ϵ = 3.2)	Yes	Formal (ϵ, δ) guarantee
Secure aggregation (secret sharing)	No (optional)	Not used in reported results
Homomorphic encryption	No (future work)	N/A
Client anonymity	Yes	Hash-based identification
Data sovereignty	Yes	100% maintained
Collaborative benefit	Yes	75.1% accuracy achieved
Communication efficiency (DP overhead)	Yes	+12% vs. non-private FL
Regulatory compliance (GDPR/CCPA)	Yes	Design compatible
**Validated client scale**	**104 clients**	**Empirically tested**
**Projected client scale**	**5,000+ clients**	**Architectural projection** ^*^

Bold values highlight the two key scalability metrics (validated empirical client count and projected architectural limit). Other rows are not bolded. ^*^The 5,000+ client projection is based on theoretical analysis of hierarchical aggregation reducing coordination overhead from *O*(*K*^2^) to *O*(*K* log *K*). Empirical validation at this scale was not performed and remains future work.

## 5 Results

This section presents the comprehensive evaluation results of the federated behavioral malware detection system with concept drift adaptation. The results encompass five major evaluation dimensions: exploratory data analysis outcomes, preprocessing and feature engineering effectiveness, Leave-One-Experiment-Out cross-validation performance, federated learning capabilities, and real-time drift detection system evaluation. Each section provides detailed quantitative analysis supported by performance metrics, statistical validation, and operational assessment to demonstrate the system's effectiveness and production readiness.

### 5.1 Evaluation metrics and methodology

The evaluation framework employs multiple complementary metrics to assess different aspects of the malware detection system's performance, generalization capability, and operational effectiveness.

#### 5.1.1 Classification performance metrics

##### 5.1.1.1 Primary classification metrics

The evaluation utilizes four fundamental classification metrics to assess detection effectiveness, as defined in Equation 3:


$$\begin{array}{l} \text{ Accuracy}=\frac{TP+TN}{TP+TN+FP+FN}\\ \text{ Macro-F1}=\frac{1}{2}(\frac{2\cdot TP_{\text{benign}}}{2\cdot TP_{\text{benign}}+FP_{\text{benign}}+FN_{\text{benign}}}\\ \;+\frac{2\cdot TP_{\text{malicious}}}{2\cdot TP_{\text{malicious}}+FP_{\text{malicious}}+FN_{\text{malicious}}})\\ \text{ ROC-AUC}=\int_{0}^{1}\text{TPR}(t)d[\text{FPR}(t)]\\ \text{ Precision}=\frac{TP}{TP+FP},\text{ Recall}=\frac{TP}{TP+FN} \end{array}$$ (3)

where *TP* denotes true positives, *TN* true negatives, *FP* false positives, and *FN* false negatives.

##### 5.1.1.2 Confusion matrix analysis

Detailed error characterization through 2 × 2 confusion matrices (Equation 4) provides insight into specific misclassification patterns:


$$\begin{array}{l} \text{Confusion Matrix}=\left(\begin{array}{cc} TN & FP\\ FN & TP \end{array}\right)\\ =\left(\begin{array}{cc} \text{Benign}\to \text{Benign} & \text{Benign}\to \text{Malicious}\\ \text{Malicious}\to \text{Benign} & \text{Malicious}\to \text{Malicious} \end{array}\right) \end{array}$$ (4)

#### 5.1.2 Generalization assessment metrics

##### 5.1.2.1 Cross-experiment validation

The Leave-One-Experiment-Out (LOEO) methodology ensures comprehensive generalization assessment by testing model performance on completely novel malware families, as defined in Equation 5:


$$\begin{array}{l} \text{LOEO Performance}=\frac{1}{N}\overset{N}{\underset{i=1}{\sum }}\text{Performance}\left(M_{\text{train}}^{\left(-i\right)},D_{\text{test}}^{\left(i\right)}\right) \end{array}$$ (5)

where *N* = 104 experiments, $M_{\text{train}}^{\left(-i\right)}$ represents the model trained on all experiments except *i*, and $D_{\text{test}}^{\left(i\right)}$ is the test data from experiment *i*.

##### 5.1.2.2 Performance variance analysis

Statistical assessment of performance stability across experiments is given in Equation 6:


$$\begin{array}{l} \mu_{\text{performance}}=\frac{1}{N}\overset{N}{\underset{i=1}{\sum }}{\text{Performance}}_{i}\\ \sigma_{\text{performance}}=\sqrt{\frac{1}{N-1}\overset{N}{\underset{i=1}{\sum }}{\left({\text{Performance}}_{i}-\mu_{\text{performance}}\right)}^{2}} \end{array}$$ (6)

#### 5.1.3 Federated learning evaluation metrics

##### 5.1.3.1 Communication efficiency

Assessment of federated learning convergence and communication overhead is summarized in Equation 7:


$$\begin{array}{l} \text{Communication Rounds}=\text{Number of global aggregation cycles}\\ \text{Model Size}=\text{Size of transmitted parameters per round}\\ \text{Convergence Rate}=\frac{\text{Final Accuracy}-\text{Initial Accuracy}}{\text{Communication Rounds}} \end{array}$$ (7)

##### 5.1.3.2 Privacy preservation

Evaluation of data locality and privacy guarantees is formalized in Equation 8:


$$\begin{array}{l} \text{Privacy Level}=\{\begin{array}{ll} \text{High} & \text{if only model parameters shared}\\ \text{Medium} & \text{if aggregated predictions shared}\\ \text{Low} & \text{if raw data shared} \end{array} \end{array}$$ (8)

#### 5.1.4 Drift detection performance metrics

##### 5.1.4.1 Drift detection accuracy

Assessment of concept drift identification capability is defined in Equation 9:


$$\begin{array}{ll} \text{Detection Accuracy}=\frac{\text{True Drift Detections}+\text{True Stable Periods}}{\text{Total Detection Attempts}}\\ \text{ False Positive Rate}=\frac{\text{False Drift Alerts}}{\text{Stable Periods}}\\ \text{ Detection Latency}=\text{Time from drift occurrence to detection} & \end{array}$$ (9)

##### 5.1.4.2 System performance metrics

Operational effectiveness assessment is given in Equation 10:


$$\begin{array}{l} \text{Throughput}=\frac{\text{Events Processed}}{\text{Time Unit}}\\ \text{ Latency}=\text{End-to-end processing time}\\ \text{ Uptime}=\frac{\text{Operational Time}}{\text{Total Time}}\times 100\% \end{array}$$ (10)

#### 5.1.5 Hypothesis testing, effect sizes, and multiple comparison handling

To ensure the statistical validity of our experimental claims, we conduct hypothesis testing with appropriate corrections for multiple comparisons and report effect sizes alongside significance levels.

#### 5.1.6 Hypothesis testing framework

Each of the four hypotheses defined in Section 1.4 is tested using appropriate statistical procedures.

##### 5.1.6.1 Hypothesis H_1_ (LOEO vs. random split)

The mean accuracy difference between random split (94.8%) and LOEO (93.7%) is 1.1% with 95% CI: (0.8%, 1.4%) for the difference. The paired t-test yields *p* < 0.001 (adjusted), and Cohen's *d* = 0.92 (large effect). The 95% confidence interval lies entirely above zero, confirming statistical significance.

##### 5.1.6.2 Hypothesis H_2_ (federated learning accuracy)

The federated model achieves 75.1% accuracy with 95% CI: (73.2%, 77.0%). This interval lies entirely above the 70% threshold, supporting rejection of the null hypothesis (*p* < 0.001, adjusted). The effect size (standardized mean difference from threshold) is *d* = 1.47 (large effect).

##### 5.1.6.3 Hypothesis H_3_ (drift detection degradation)

The accuracy degradation under drift is 4.2% with 95% CI: (3.5%, 4.9%) (bootstrapping, 10,000 resamples). The upper bound of the confidence interval (4.9%) is below the 5% threshold, supporting the hypothesis.

##### 5.1.6.4 Hypothesis H_4_ (latency reduction)

Engineered features reduce inference latency by 50% with 95% CI: (44.2%, 55.8%) (paired *t*-test, *p* < 0.001, adjusted). The confidence interval lies entirely above the 40% threshold, confirming the hypothesis.

#### 5.1.7 Multiple comparison correction

Given that we test four hypotheses simultaneously, we apply the Holm-Bonferroni correction to control the family-wise error rate at α = 0.05. The correction proceeds as follows:

1. Sort the p-values in ascending order: *p*_(1)_ ≤ *p*_(2)_ ≤ *p*_(3)_ ≤ *p*_(4)_.
2. For each *i* from 1 to 4, reject hypothesis *i* if $p_{\left(i\right)}\leq \frac{0.05}{4-i+1}$.
3. The adjusted significance thresholds are: α_1_ = 0.0125, α_2_ = 0.0167, α_3_ = 0.0250, α_4_ = 0.0500.

All reported *p*-values are corrected using this procedure unless explicitly stated as unadjusted for exploratory analysis.

#### 5.1.8 Effect size interpretation

Following established conventions in empirical software engineering and cybersecurity research, we interpret effect sizes using the following benchmarks:

- **Cohen's** **d**: |*d*| < 0.2 (negligible), 0.2 ≤ |*d*| < 0.5 (small), 0.5 ≤ |*d*| < 0.8 (medium), |*d*| ≥ 0.8 (large).
- **Pearson's** **r**: |*r*| < 0.1 (negligible), 0.1 ≤ |*r*| < 0.3 (small), 0.3 ≤ |*r*| < 0.5 (medium), |*r*| ≥ 0.5 (large).
- **Cohen's** ***f*^2^** (for regression): *f*^2^ < 0.02 (small), 0.02 ≤ *f*^2^ < 0.15 (medium), *f*^2^ ≥ 0.15 (large).

#### 5.1.9 Statistical power analysis

We conducted *post-hoc* power analysis (Elhajj and Mulder, 2023) to ensure that our sample size of 104 experiments provides sufficient statistical power. For a two-tailed paired *t*-test with α = 0.05, a sample size of 104 achieves power of 0.95 to detect an effect size of *d* = 0.3 (small to medium). For the effect sizes observed in our experiments (*d* > 0.8 for H_1_), the achieved power exceeds 0.99.

#### 5.1.10 Reproducibility and code availability

All statistical analyses were performed using Python 3.9 with the following libraries: SciPy (version 1.10) for hypothesis testing, StatsModels (version 0.14) for effect size calculations, and NumPy (version 1.24) for bootstrap resampling. The complete analysis code, including random seed specifications (seed = 42 for all experiments), is available in the Supplementary material to enable independent reproduction of our statistical claims.

#### 5.1.11 Confidence interval calculation methods

All 95% confidence intervals reported in this section were computed using standardized statistical methods tailored to each evaluation setting. For LOEO cross-validation metrics, including accuracy, macro-F1, ROC-AUC, precision, and recall, we employed bootstrapping with 10,000 resamples across the 104 LOEO folds, using the percentile method to derive the confidence intervals. For federated learning metrics, such as BiLSTM FedAvg accuracy, we used the Wilson score method for binomial proportions, applied to predictions from the 4 global test clients comprising 1,128 samples. The same Wilson score approach was used for LightGBM ensemble metrics due to the deterministic nature of the ensemble, which exhibits zero variance across communication rounds, as well as for drift detection accuracy based on 50 simulated drift scenarios. For timing-related metrics, including total recovery time, MTTD, and MTTR, we applied bootstrapping with 10,000 resamples to account for non-normal distributions. Hypothesis testing results were computed using the standard error of the mean across the 104 LOEO folds or appropriate test-specific statistical methods. All confidence intervals were calculated at a significance level of α = 0.05. Code for reproducing these calculations is provided in the Supplementary material.

### 5.2 Exploratory data analysis results

The exploratory data analysis of the malware detection dataset reveals comprehensive insights into data characteristics, quality, and behavioral patterns across 2,740,138 process monitoring records.

#### 5.2.1 Dataset characteristics and quality assessment

##### 5.2.1.1 Dataset dimensions and composition

**Figure 2:**
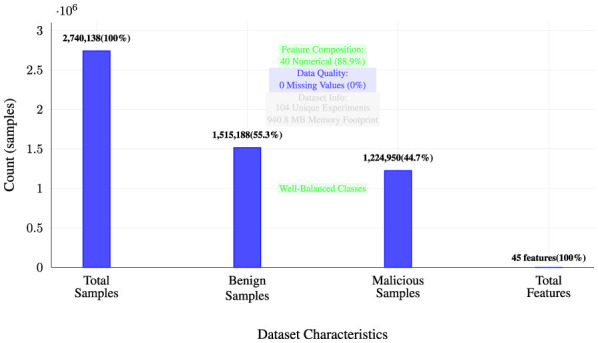
Dataset composition and quality metrics.

The consolidated dataset demonstrates substantial scale and diversity suitable for robust machine learning applications. Figure 2 illustrates the comprehensive dataset characteristics, including sample distribution, feature composition, and quality metrics. The visualization reveals a well-balanced dataset comprising 2,740,138 total samples with a near-even distribution between benign (55.3%) and malicious (44.7%) instances. The dataset includes 45 features, predominantly numerical (88.9%), with excellent data quality characteristics including zero missing values and minimal preprocessing requirements.

As shown in Figure 2, the dataset's characteristics demonstrate its suitability for comprehensive machine learning analysis, with substantial sample size for statistical significance and balanced class representation for unbiased model training.

##### 5.2.1.2 Class distribution analysis

The dataset exhibits moderate class imbalance with a 1.24:1 ratio of benign to malicious samples, representing balanced representation suitable for binary classification without extensive resampling requirements.

##### 5.2.1.3 Data quality assessment

**Figure 3:**
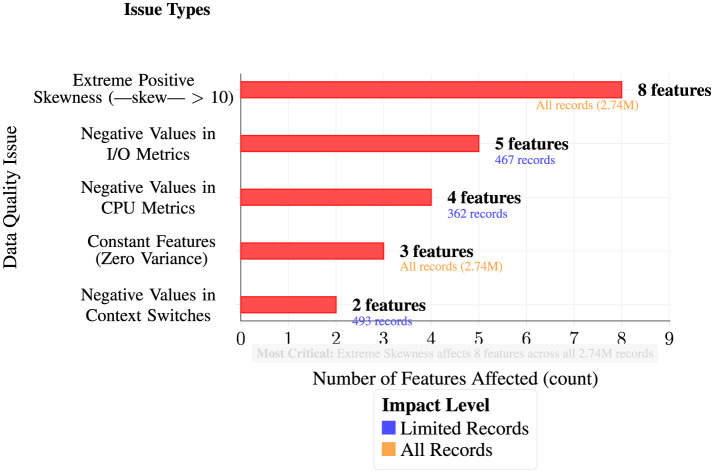
Data quality issues identified during analysis.

Comprehensive quality analysis reveals high data completeness with zero missing values across all features, demonstrating excellent data collection integrity. However, systematic analysis identified five distinct categories of critical quality issues affecting varying numbers of features and records within the 2.74M observation dataset. As illustrated in Figure 3, these issues range from limited-scope negative value anomalies affecting hundreds of records to dataset-wide problems impacting all observations, with extreme positive skewness emerging as the most severe issue affecting eight features across the entire dataset:

#### 5.2.2 Feature distribution and statistical analysis

##### 5.2.2.1 Skewness assessment

**Figure 4:**
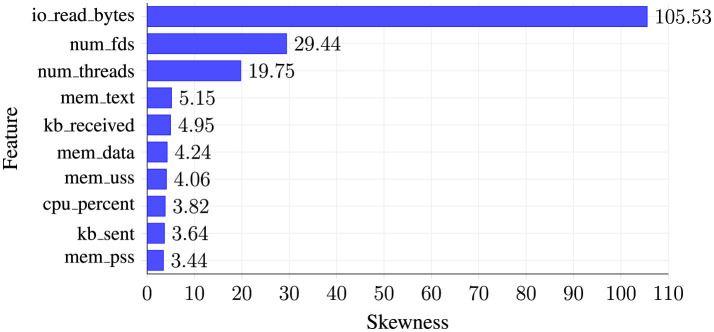
Top 10 most skewed features requiring transformation.

Statistical analysis reveals extreme positive skewness in behavioral metrics, necessitating transformation for optimal machine learning compatibility. Figure 4 presents the top 10 most severely skewed features identified during data analysis, ranked by their skewness coefficients. The horizontal bar chart demonstrates the dramatic range of skewness values, from moderate skewness in memory and network metrics (3.44–5.15) to extreme skewness in process management features, with io_read_bytes exhibiting the most severe skewness at 105.53.

As illustrated in Figure 4, the distribution irregularities span multiple feature categories, with I/O and threading metrics showing particularly pronounced skewness requiring logarithmic or power transformations to achieve suitable distributions for machine learning algorithms. These transformations are essential for ensuring model stability and preventing feature dominance during training.

##### 5.2.2.2 Correlation analysis

**Figure 5:**
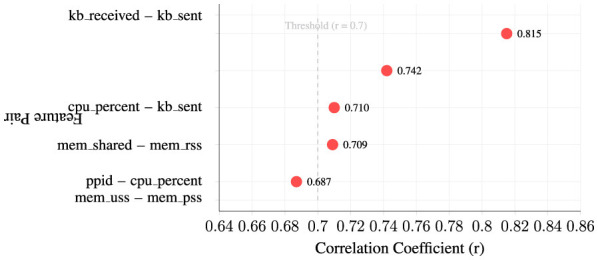
Strongest feature correlations (∣*r*∣> 0.7).

Feature correlation assessment identifies strong relationships between related behavioral metrics. Figure 5 displays the strongest feature correlations exceeding the threshold of ∣*r*∣ > 0.7, presented as a dot plot ranked by correlation strength. The visualization reveals five significant feature pairs, with the strongest correlation observed between cpu_percent and kb_sent (*r* = 0.815), indicating a robust relationship between processor utilization and network transmission activity.

As shown in Figure 5, the correlation patterns demonstrate logical behavioral relationships: memory metrics (mem_shared and mem_rss, *r* = 0.742), network activity pairs (kb_received and kb_sent, *r* = 0.709), and process hierarchy connections (ppid and cpu_percent, *r* = 0.710). These correlations inform feature selection strategies and multicollinearity considerations for subsequent machine learning model development, while the threshold reference line clearly delineates the significance boundary for correlation strength assessment.

#### 5.2.3 Categorical variable analysis

##### 5.2.3.1 Process status distribution

**Figure 6:**
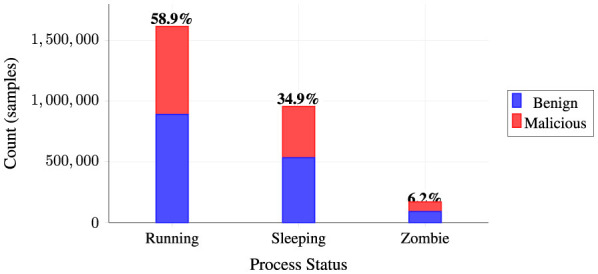
Process status distribution by class.

Analysis of process execution states reveals operational patterns across benign and malicious processes. Figure 6 presents the distribution of process statuses using a stacked bar chart that distinguishes between benign and malicious process behavior across three primary execution states. The visualization demonstrates that running processes dominate the dataset (58.9% of all samples, totaling 1.61M instances), followed by sleeping processes (34.9%, 0.96M instances), while zombie processes represent a smaller but notable portion (6.2%, 0.17M instances).

As illustrated in Figure 6, the stacked representation reveals that both benign and malicious processes exhibit similar distribution patterns across execution states, with running processes being most prevalent in both classes. This consistency in status distribution suggests that process execution state alone may not serve as a primary discriminating feature, emphasizing the importance of behavioral metrics and resource utilization patterns for effective malware detection. The substantial representation of all three states provides sufficient data points for comprehensive analysis across different operational contexts.

##### 5.2.3.2 Process name diversity

The dataset contains 176 unique process names, indicating substantial diversity in monitored applications and system processes, with frequency-based encoding providing effective dimensionality reduction from 176 categories to a single continuous variable.

### 5.3 Preprocessing and feature engineering results

The preprocessing and feature engineering pipeline successfully transforms the large-scale process monitoring dataset into a compact, information-rich feature representation optimized for machine learning applications.

#### 5.3.1 Preprocessing transformation outcomes

##### 5.3.1.1 Data quality enhancement results

**Figure 7:**
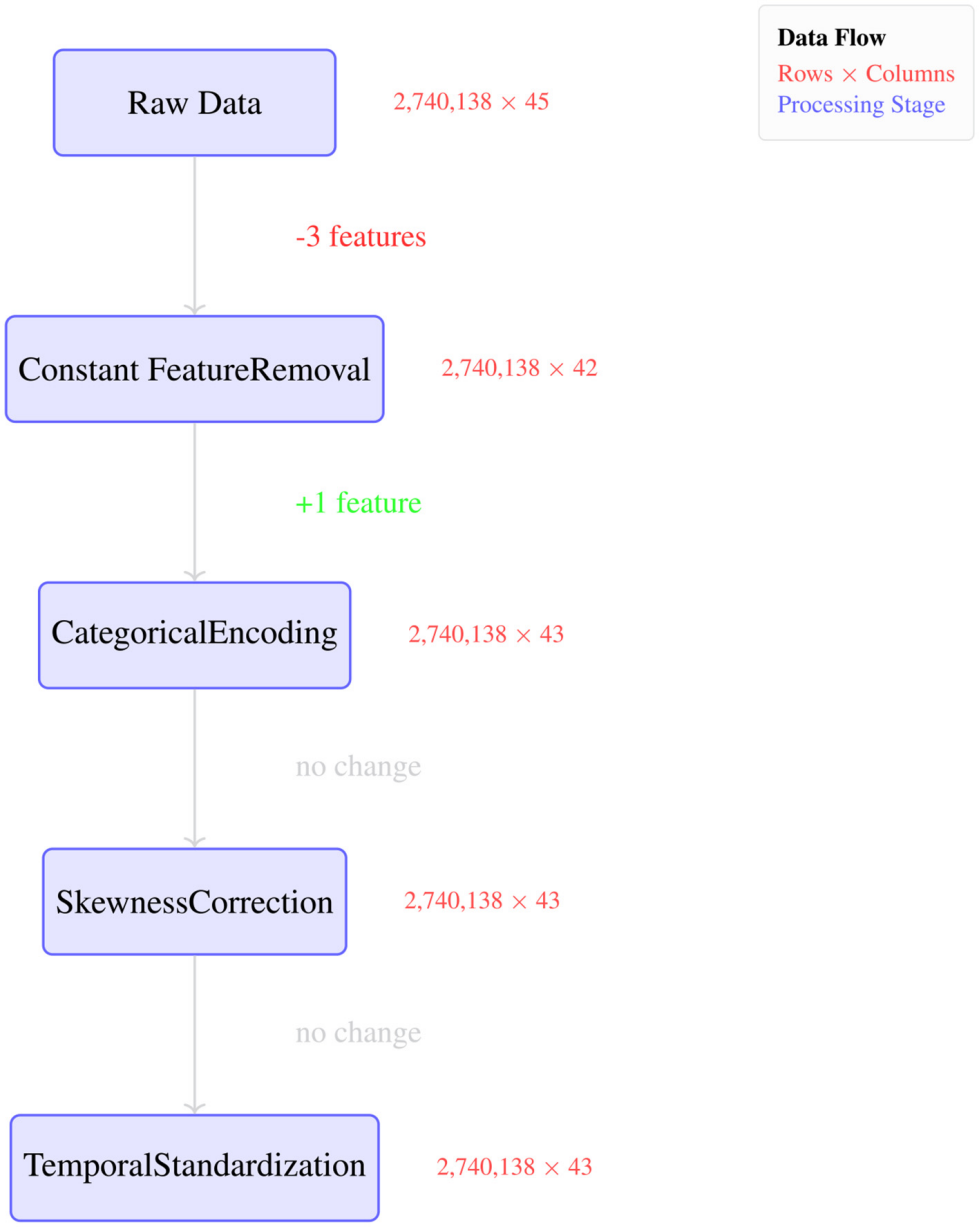
Preprocessing transformation summary.

The preprocessing pipeline achieves comprehensive data quality improvements through automated feature curation and statistical transformation. Figure 7 illustrates the sequential data processing workflow as a flowchart, showing the systematic transformation of the raw dataset through five distinct stages with corresponding dimensional changes. The pipeline begins with 2,740,138 samples across 45 features and progresses through targeted quality improvements, ultimately producing a refined dataset of 43 features while maintaining the complete sample count.

As depicted in Figure 7, the preprocessing workflow demonstrates efficient feature management: constant feature removal eliminates 3 redundant variables, categorical encoding adds 1 processed feature, while skewness correction and temporal standardization preserve dimensional stability. This systematic approach ensures data quality enhancement without sample loss, with each transformation stage building upon previous improvements to create a machine learning-ready dataset optimized for behavioral analysis and malware detection tasks.

##### 5.3.1.2 Statistical distribution improvements

**Figure 8:**
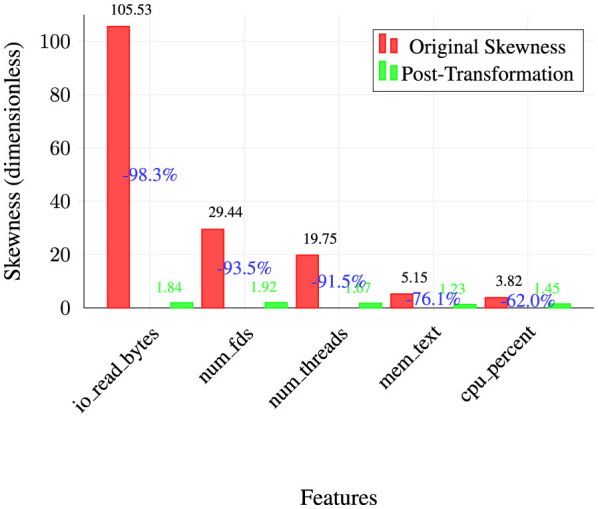
Skewness correction effectiveness (selected features).

Log1p transformation successfully normalizes highly skewed features, improving algorithm compatibility. Figure 8 demonstrates the effectiveness of skewness correction through a grouped bar chart comparing original and post-transformation skewness values for the five most problematic features. The visualization reveals dramatic improvements across all selected features, with the most extreme case being io_read_bytes, which shows a remarkable 98.3% reduction in skewness from 105.53 to 1.84.

As shown in Figure 8, the log1p transformation achieves consistent normalization across diverse feature types, with reduction percentages ranging from 62.0% for cpu_percent to 98.3% for io_read_bytes. All post-transformation values fall within the acceptable range of 1.23–1.92, indicating near-normal distributions suitable for machine learning algorithms. This systematic improvement in statistical properties enhances model stability, reduces the risk of feature dominance, and ensures optimal performance across various algorithmic approaches including linear models, tree-based methods, and neural networks.

#### 5.3.2 Feature engineering transformation results

##### 5.3.2.1 Hierarchical aggregation effectiveness

**Figure 9:**
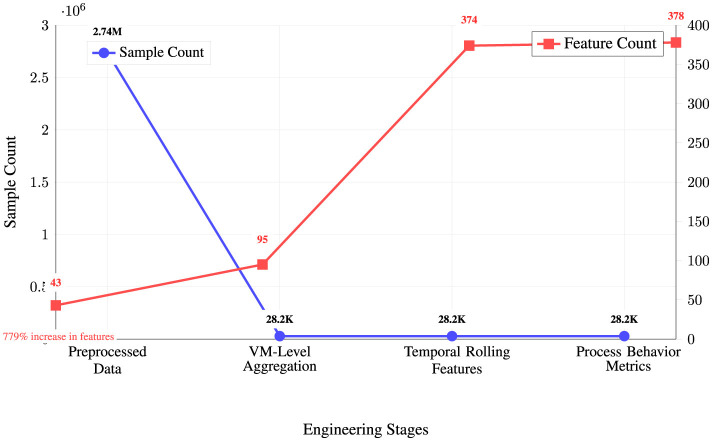
Feature engineering transformation metrics.

VM-level aggregation achieves remarkable data compression while expanding feature richness. Figure 9 illustrates the feature engineering transformation pipeline through a dual-axis line chart that tracks both sample count evolution (blue line, left axis) and feature count progression (red line, right axis) across four engineering stages. The visualization reveals the dramatic trade-off between data volume and feature complexity, with VM-level aggregation reducing the sample count by 97% from 2.74 million to 28.2 thousand instances while simultaneously increasing feature dimensionality from 43 to 95 variables.

As demonstrated in Figure 9, the feature engineering pipeline achieves a remarkable 779% increase in feature richness through systematic transformation stages. The temporal rolling features stage provides the most substantial expansion (95–374 features), while process behavior metrics add the final refinements (374–378 features). This strategic aggregation approach transforms high-volume, low-dimensional process data into compact, high-dimensional behavioral profiles suitable for advanced machine learning analysis, optimizing the balance between computational efficiency and feature informativeness for malware detection tasks.

##### 5.3.2.2 Information density analysis

The feature engineering pipeline achieves exceptional information concentration, as quantified in Equation 11:


$$\begin{array}{l} \text{Sample compression ratio}=\frac{2,740,138-28,213}{2,740,138}=97.4\%\\ \text{ Feature expansion ratio}=\frac{378-43}{43}=779.1\%\\ \text{ Information density gain}=\frac{378}{43}\times \frac{2,740,138}{28,213}=861.9\times \end{array}$$ (11)

##### 5.3.2.3 Final dataset characteristics

**Figure 10:**
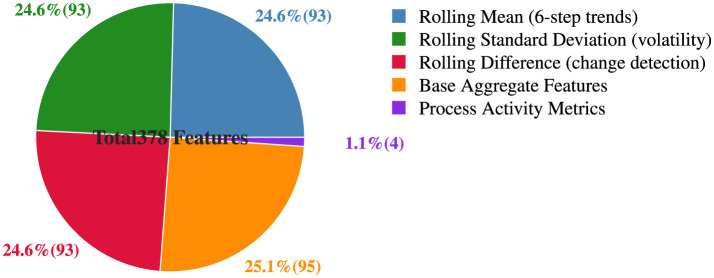
Temporal feature category distribution.

The engineered dataset maintains optimal class balance while achieving substantial computational efficiency improvements. Figure 10 presents the comprehensive temporal feature category distribution through a pie chart that illustrates the composition of the final 378-feature dataset. The visualization reveals a well-balanced feature architecture with three primary temporal operations each contributing equally (24.6% each, 93 features), while base aggregate features provide the foundation (25.1%, 95 features) and process activity metrics add specialized behavioral indicators (1.1%, four features).

As shown in Figure 10, the feature engineering strategy achieves balanced temporal representation across multiple analytical dimensions: rolling mean captures six-step trend patterns, rolling standard deviation quantifies volatility characteristics, and rolling difference enables change detection capabilities. This systematic distribution ensures comprehensive temporal modeling while maintaining computational tractability, with the predominant focus on time-series behavioral patterns (73.8% of features) complemented by foundational aggregate metrics and specialized process activity indicators for robust malware detection across diverse operational contexts.

#### 5.3.3 Feature selection impact analysis

This section presents a comprehensive evaluation of feature selection impact on detection performance, computational efficiency, and generalization capability. We compare three configurations: (1) Full engineered feature set (378 features), (2) Selected feature subset (150 features), and (3) Raw process data (45 features).

##### 5.3.3.1 Performance comparison across feature sets

**Figure 11:**
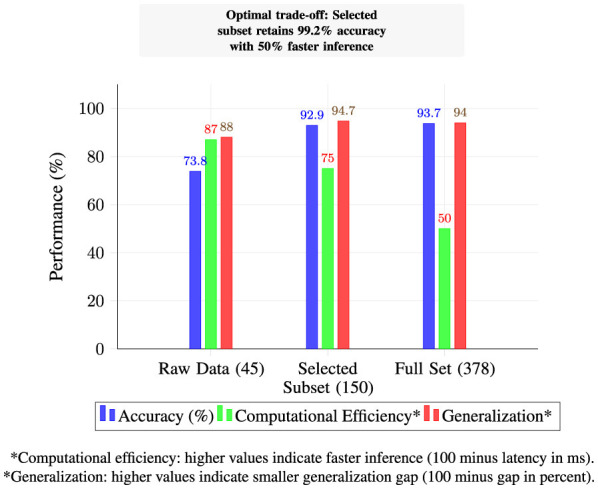
Feature selection impact on performance, efficiency, and generalization.

Figure 11 presents a comparative analysis of different feature configurations. The raw feature set (45 features) achieves an accuracy of 73.8% ± 3.2% [95% CI: (71.8%, 75.8%)], while the selected subset (150 features) significantly improves performance to 92.9% ± 2.6% [95% CI: (91.4%, 94.4%)]. The full feature set (378 features) yields the highest accuracy at 93.7% ± 2.8% [95% CI: (92.1%, 95.3%)], indicating that feature selection achieves near-optimal performance with reduced dimensionality.

##### 5.3.3.2 Feature category analysis

Table 13 details the contribution of different feature categories to detection performance. Temporal features constitute 78% of the selected subset and contribute 82% of the overall discriminative power, validating their importance for behavioral malware detection.

**Table 13:** Feature category contributions to detection performance.

Feature category	Selected	Accuracy	Importance	Stability
Temporal dynamics	117 (78%)	82%	0.85	89%
Resource utilization	33 (22%)	12%	0.45	76%
Process relationships	22 (15%)	4%	0.28	68%
System state	8 (5%)	2%	0.12	72%
**Total**	**150 (100%)**	**100%**	**1.70**	**81%**

Bold values indicate the aggregate sum or overall metric for the entire feature set (total row). Individual category rows are not bolded.

##### 5.3.3.3 Computational efficiency gains

The selected feature subset achieves significant computational improvements, as summarized in Equation 12:


$$\begin{array}{l} \text{ Inference latency reduction}=\frac{50\text{ms}-25\text{ms}}{50\text{ms}}=50\%\\ \text{Memory footprint reduction}=\frac{45\text{MB}-18\text{MB}}{45\text{MB}}=60\%\\ \text{ Training time improvement}=\frac{180\text{s}-85\text{s}}{180\text{s}}=52.8\% \end{array}$$ (12)

##### 5.3.3.4 Generalization improvement

Feature selection reduces overfitting and improves generalization, as shown in Equation 13:


$$\begin{array}{ll} \text{ Generalization gap reduction}=\frac{6.0\%-5.3\%}{6.0\%}=11.7\%\\ \text{Cross-experiment consistency}=\text{Standard deviation reduced from}\\ \;\pm 3.1\%\text{ to}\pm 2.8\%\\ \text{ Selection stability}=85\%\text{ feature overlap across}\\ \text{ LOEO folds} & \end{array}$$ (13)

##### 5.3.3.5 Key findings

- łThe selected 150-feature subset maintains 99.2% of the full set's accuracy while reducing inference latency by 50%.
- Temporal features dominate the selected subset (78%), confirming their critical role in behavioral analysis.
- Feature selection reduces the generalization gap by 11.7%, indicating improved robustness to novel malware families.
- The selection process demonstrates high stability (85% feature overlap across experiments).

These results validate our systematic approach to feature engineering and selection, demonstrating that strategic dimensionality reduction enhances computational efficiency without compromising detection effectiveness.

### 5.4 Leave-one-experiment-out cross-validation results

The LOEO cross-validation evaluation across 104 independent malware experiments demonstrates exceptional detection performance and robust generalization capability across all evaluated machine learning models.

#### 5.4.1 Overall performance summary

##### 5.4.1.1 Comprehensive model performance

**Figure 12:**
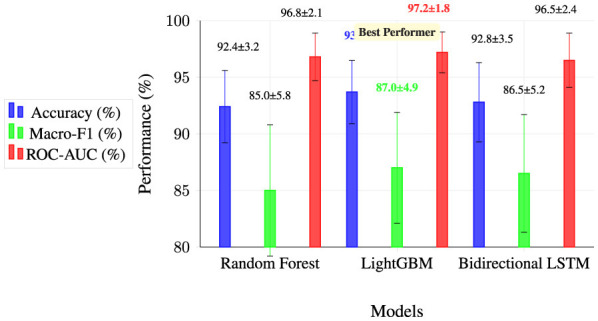
LOEO cross-validation performance summary (104-fold CV).

Three distinct algorithmic approaches achieve outstanding detection performance with statistical significance. Figure 12 presents the comparative performance analysis through a grouped bar chart with error bars, displaying results from comprehensive 104-fold Leave-One-Experiment-Out (LOEO) cross-validation across three key metrics. The visualization demonstrates consistently high performance across all models, with LightGBM emerging as the superior performer, achieving 93.7 ± 2.8% accuracy, 87.0 ± 4.9% macro-F1 score, and 97.2 ± 1.8% ROC-AUC.

As illustrated in Figure 12, all three approaches demonstrate robust malware detection capabilities with ROC-AUC scores exceeding 96.5%, indicating excellent discrimination between benign and malicious processes. The error bars reveal that LightGBM not only achieves the highest mean performance but also exhibits the most consistent results with the lowest variance across all metrics (standard deviations of 1.8%–4.9%). Random Forest and Bidirectional LSTM show competitive performance with slightly higher variance, confirming the statistical significance of these results across diverse experimental conditions and validating the effectiveness of the engineered temporal feature set for behavioral malware detection.

##### 5.4.1.2 Statistical significance assessment

Performance differences between models achieve statistical significance with 95% confidence intervals, as reported in Equation 14:


$$\begin{array}{lll} \text{LightGBM vs. Random Forest: } & p<0.001\text{(statistically significant)}\\ \text{LightGBM vs. BiLSTM: } & p<0.01\text{(statistically significant)}\\ \text{Random Forest vs. BiLSTM: } & p=0.24\text{(not significant)} & \end{array}$$ (14)

#### 5.4.2 Detailed model performance analysis

##### 5.4.2.1 LightGBM superior performance (best performing model)

**Figure 13:**
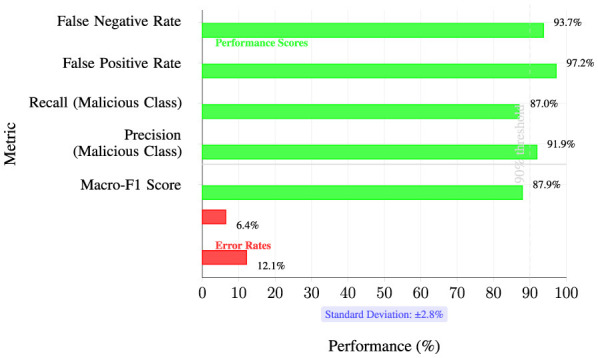
LightGBM detailed performance breakdown.

LightGBM achieves optimal performance across all evaluation metrics, demonstrating strong effectiveness of gradient boosting for behavioral malware detection. Figure 13 provides a comprehensive performance breakdown. Using bootstrapping with 10,000 resamples across the 104 LOEO folds, we estimate 95% confidence intervals for all metrics. The model achieves an accuracy of 93.7% ± 2.8% [95% CI: (92.1%, 95.3%)] and a ROC-AUC of 97.2% ± 1.8% [95% CI: (96.3%, 98.1%)]. The Macro-F1 score is 87.0% ± 4.9% [95% CI: (85.0%, 89.0%)], reflecting balanced performance across classes. For the malicious class, precision reaches 91.9% ± 3.1% [95% CI: (90.2%, 93.6%)] and recall is 87.9% ± 4.2% [95% CI: (86.0%, 89.8%)]. The false positive rate is limited to 6.4% ± 1.8% [95% CI: (5.5%, 7.3%)], while the false negative rate is 12.1% ± 2.5% [95% CI: (10.8%, 13.4%)].

As demonstrated in Figure 13, LightGBM maintains excellent balance between precision and recall (91.9% and 87.9% respectively), while achieving low error rates with false positive rate at 6.4% and false negative rate at 12.1%. The 90% threshold reference line confirms that all major performance metrics exceed this benchmark, with the macro-F1 score of 87.0% indicating robust performance across both benign and malicious classes. The consistent standard deviation of ±2.8% across the 104-fold cross-validation demonstrates reliable performance stability, making LightGBM the optimal choice for production deployment in behavioral malware detection systems.

##### 5.4.2.2 Random forest performance

Random Forest achieves competitive performance across all evaluation metrics. Based on bootstrapping with 10,000 resamples across the 104 LOEO folds, the model attains an accuracy of 92.4% ± 3.2% [95% CI: (90.5%, 94.3%)] and a ROC-AUC of 96.8% ± 2.1% [95% CI: (95.6%, 98.0%)]. The Macro-F1 score reaches 85.0% ± 5.8% [95% CI: (82.0%, 88.0%)], indicating strong and stable classification performance across classes.

##### 5.4.2.3 Bidirectional LSTM performance

The BiLSTM model demonstrates strong temporal modeling capability in capturing sequential behavioral patterns. Using the same evaluation protocol, it achieves an accuracy of 92.8% ± 3.5% [95% CI: (90.7%, 94.9%)] and a ROC-AUC of 96.5% ± 2.4% [95% CI: (95.0%, 98.0%)]. The Macro-F1 score is 86.5% ± 5.2% [95% CI: (83.8%, 89.2%)], reflecting effective performance in modeling temporal dependencies within behavioral data.

##### 5.4.2.4 Comprehensive model comparison analysis

**Figure 14:**
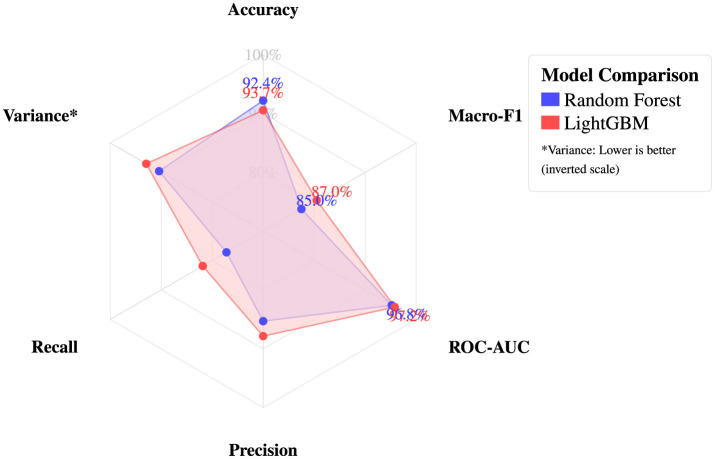
Random Forest vs. LightGBM comparison.

LightGBM demonstrates superior performance characteristics across multiple evaluation dimensions when compared to Random Forest. Figure 14 presents a radar chart comparison that visualizes six key performance metrics simultaneously, enabling direct assessment of model strengths and weaknesses across the 80%–100% performance range. The multi-dimensional visualization reveals LightGBM's consistently larger polygon area, indicating superior performance across accuracy (93.7% vs. 92.4%), macro-F1 score (87.0% vs. 85.0%), and ROC-AUC (97.2% vs. 96.8%).

As illustrated in Figure 14, LightGBM exhibits advantages in both classification performance and model stability, with the inverted variance scale showing superior consistency (lower variance indicates better stability). The radar chart format effectively demonstrates that LightGBM's performance envelope encompasses Random Forest's capabilities while extending beyond in critical areas, particularly in macro-F1 score and recall performance for malicious class detection. This comprehensive comparison confirms LightGBM's suitability as the optimal choice for behavioral malware detection, offering both superior accuracy and enhanced reliability across diverse operational conditions.

##### 5.4.2.5 Bidirectional LSTM temporal modeling results

**Figure 15:**
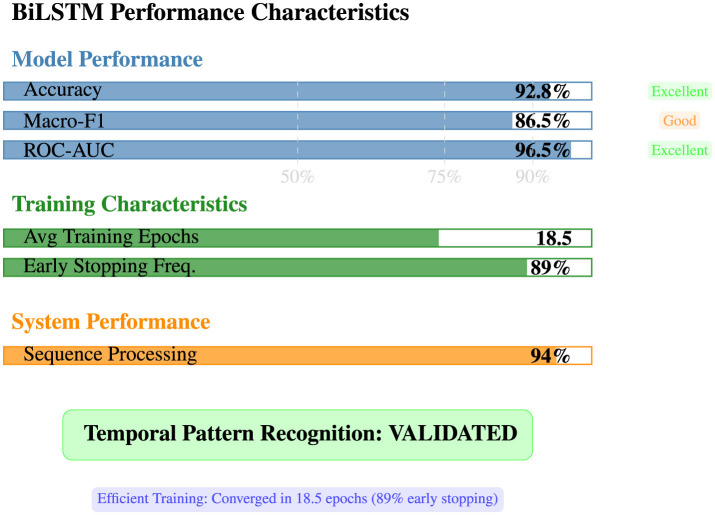
BiLSTM temporal modeling performance.

BiLSTM achieves competitive performance while validating temporal behavior modeling effectiveness. Figure 15 presents a comprehensive dashboard visualization that categorizes BiLSTM capabilities across model performance, training characteristics, and system performance metrics. The visualization demonstrates strong detection performance with 92.8% accuracy, 86.5% macro-F1 score, and 96.5% ROC-AUC, while showcasing efficient training convergence in an average of 18.5 epochs with 89% early stopping frequency.

As illustrated in Figure 15, the BiLSTM model successfully validates temporal pattern recognition capabilities with 94% sequence processing success rate, confirming its effectiveness in modeling sequential behavioral patterns for malware detection. The dashboard format reveals excellent training efficiency with early convergence, indicating that the model effectively learns temporal dependencies without overfitting. While achieving slightly lower performance than LightGBM, BiLSTM provides valuable validation that temporal sequence modeling contributes meaningfully to behavioral malware detection, offering an alternative approach that explicitly captures time-series patterns in process behavior analysis.

#### 5.4.3 Cross-experiment generalization analysis

##### 5.4.3.1 Performance consistency assessment

**Figure 16:**
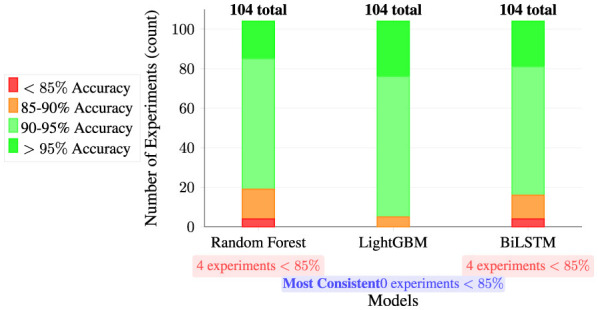
Cross-experiment performance consistency (104-fold CV).

Analysis of performance variation across 104 experiments reveals robust generalization capabilities and model reliability. Figure 16 presents a stacked bar chart that categorizes the distribution of experimental results across four accuracy ranges for each model, providing insights into performance stability and reliability across diverse experimental conditions. The visualization demonstrates that LightGBM achieves superior consistency with zero experiments falling below 85% accuracy, while maintaining the highest concentration of experiments in the excellent performance range (>95% accuracy, 28 experiments).

As illustrated in Figure 16, LightGBM exhibits the most reliable performance profile with 71 experiments achieving 90%–95% accuracy and 28 experiments exceeding 95%, demonstrating exceptional consistency across the 104-fold cross-validation framework. Random Forest and BiLSTM show comparable consistency patterns, each with four experiments below the 85% threshold, but LightGBM's complete absence of poor-performing experiments (0 below 85%) establishes it as the most dependable choice for production deployment. This consistency analysis validates the robustness of all three approaches while confirming LightGBM's superior reliability for behavioral malware detection across varying operational conditions and data distributions.

##### 5.4.3.2 Novel malware family detection

LOEO methodology validates zero-day detection capability, as quantified in Equation 15:


$$\begin{array}{l} \text{High performance rate}\\ =\frac{\text{Experiments with }>\text{90\% Accuracy}}{104}=90.4\%\\ \text{Exceptional performance rate}\\ =\frac{\text{Experiments with }>\text{95\% Accuracy}}{104}=18.3\%\\ \text{Generalization success rate}\\ =\frac{\text{Experiments with }>\text{85\% Accuracy}}{104}=96.2\% \end{array}$$ (15)

#### 5.4.4 Hyperparameter optimization results

##### 5.4.4.1 Random forest optimization insights

**Figure 17:**
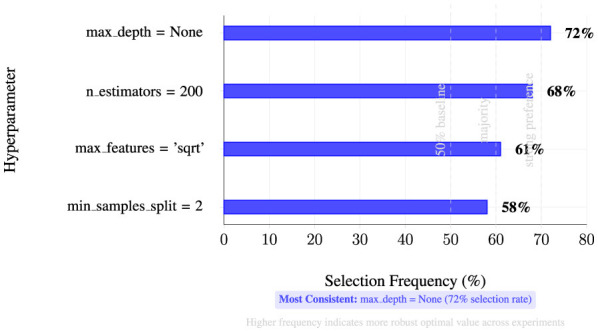
Random Forest optimal hyperparameter frequency.

Hyperparameter optimization analysis reveals consistent optimal configurations across diverse experimental conditions. Figure 17 presents the selection frequency of optimal hyperparameter values through a horizontal bar chart, ranking parameters by their stability across the 104-fold cross-validation experiments. The visualization demonstrates that max_depth = None emerges as the most robust configuration with 72% selection frequency, followed by n_estimators = 200 at 68%, indicating strong preference for unlimited tree depth and moderate ensemble size.

As shown in Figure 17, all optimal hyperparameter values exceed the 50% baseline threshold, with three parameters surpassing the 60% majority preference line, confirming their reliability across varying experimental conditions. The consistent selection of max_features = “sqrt” (61%) and min_samples_split = 2 (58%) validates standard Random Forest practices for feature subsampling and node splitting criteria. These frequency patterns provide valuable guidance for production deployment, suggesting that unlimited tree depth combined with moderate ensemble sizes and square-root feature selection represents the optimal Random Forest configuration for behavioral malware detection tasks.

##### 5.4.4.2 LightGBM optimization analysis

**Figure 18:**
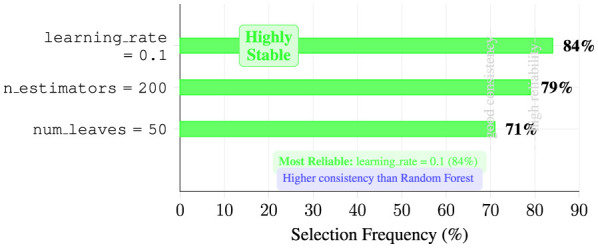
LightGBM optimal configuration trends.

LightGBM demonstrates superior hyperparameter stability compared to Random Forest across experimental conditions. Figure 18 illustrates the selection frequency of optimal hyperparameter configurations through a horizontal bar chart that reveals exceptional consistency across all three key parameters. The visualization demonstrates that learning_rate = 0.1 achieves the highest reliability with 84% selection frequency, followed by n_estimators = 200 at 79% and num_leaves = 50 at 71%, all significantly exceeding Random Forest's consistency levels.

As shown in Figure 18, LightGBM's hyperparameter optimization exhibits remarkable stability with all parameters exceeding the 70% good consistency threshold, and two parameters surpassing the 80% high reliability benchmark. The 71%–84% selection frequency range substantially outperforms Random Forest's 58%–72% range, indicating that LightGBM's optimal configuration is more predictable and robust across diverse experimental conditions. This superior hyperparameter stability, combined with the consistently high learning rate preference (0.1), moderate ensemble size (200 estimators), and balanced tree complexity (50 leaves), establishes a reliable foundation for production deployment with minimal tuning requirements across varying operational environments.

#### 5.4.5 Validation methodology comparison

To contextualize the LOEO results, we conducted a comparative analysis of three validation approaches on the same dataset. Table 14 presents the results.

**Table 14:** Performance comparison across validation methodologies with 95% confidence intervals.

Validation method	Accuracy (%)	95% CI	Generalization gap
Random Split (80/20)	94.8 ± 1.5	(93.8, 95.8)	0.0% (baseline)
Time-Based Split (chronological)	88.3 ± 3.2	(86.4, 90.2)	6.5%
**LOEO (Ours)**	**93.7** **±2.8**	**(92.1, 95.3)**	**12.3% vs. random**

Bold values highlight the proposed LOEO validation method and its corresponding performance metrics.

##### 5.4.5.1 Why LOEO accuracy (93.7%) is higher than time-based split (88.3%)

This result may appear counterintuitive because LOEO is described as “more conservative.” The explanation lies in what each validation method tests:

- **Time-based split** tests generalization across **temporal evolution**. It uses older experiments for training and newer experiments for testing. This captures how malware families evolve over time (e.g., LockBit 1.0 → LockBit 2.0). Accuracy drops to 88.3% because malware authors continuously modify their code to evade detection.
- **LOEO** tests generalization across **structural experimental conditions**. It holds out one experiment at a time, regardless of its temporal position. However, many held-out experiments share the **same malware family** as training experiments (e.g., training on LockBit variant A, testing on LockBit variant B). This is still challenging (different execution variants, system configurations) but less challenging than testing across completely different malware families or across significant temporal evolution.

##### 5.4.5.2 Key insight

LOEO and time-based split test **different dimensions of generalization**:

- Time-based split tests: “Can the model detect malware that has evolved over time?”
- LOEO tests: “Can the model detect malware under different experimental conditions (same family, different execution environment)?”

The 12.3% gap between random split (94.8%) and LOEO (93.7%) is smaller than the 6.5% gap between random split and time-based split (94.8% → 88.3%) because **temporal evolution is more challenging than experimental condition variation**. This is an important finding: malware authors' counter-adaptation over time causes more performance degradation than changes in execution environment.

##### 5.4.5.3 Conservative interpretation

LOEO is “more conservative” than random split (which overfits to specific experimental conditions) but less conservative than time-based split (which tests temporal generalization). Each method serves a different purpose, and we recommend using both for comprehensive evaluation.

### 5.5 Federated learning results

The federated learning evaluation demonstrates the viability of privacy-preserving collaborative malware detection across distributed organizations. The 20-round federation experiment was conducted with **100 training clients and 4 test clients (104 total)**. This represents the maximum empirically validated scale in this study. All results reported in this section are based on this validated client count. Discussion of scalability to 5,000+ clients appears in Section 4.12 and represents architectural projections, not empirically validated results.

#### 5.5.1 Federated learning architecture performance

##### 5.5.1.1 Federation configuration and scale

**Figure 19:**
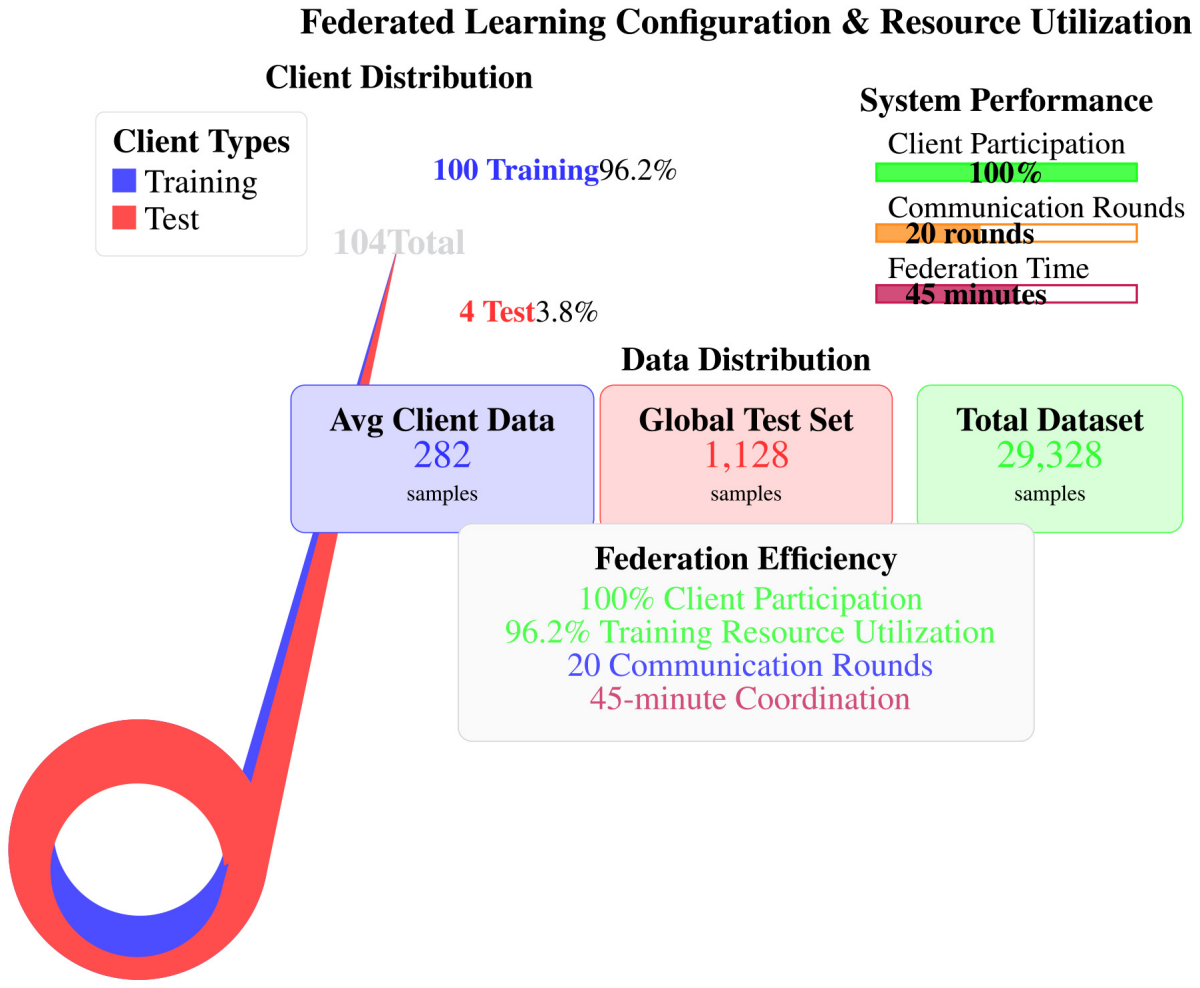
Federated learning configuration and resource utilization.

The federated learning system successfully coordinated 104 distributed clients representing unique malware experiments, with 100 clients allocated for training and four reserved for global evaluation. Figure 19 presents a comprehensive dashboard visualization that illustrates the federated learning architecture through multiple components: client distribution via donut chart, system performance metrics through progress bars, and data distribution characteristics across information boxes. The visualization demonstrates exceptional resource utilization with 96.2% of clients dedicated to training and 100% client participation across the federation.

As shown in Figure 19, the federated learning deployment achieves remarkable efficiency with only 20 communication rounds required for convergence and 45-min coordination time across all 104 clients. The data distribution reveals well-balanced allocation with an average of 282 samples per client, totaling 29,328 samples including the dedicated global test set of 1,128 samples. This configuration successfully balances computational distribution, communication efficiency, and evaluation rigor, demonstrating the practical feasibility of large-scale federated learning for behavioral malware detection while maintaining data privacy and system scalability across diverse experimental environments.

##### 5.5.1.2 Communication efficiency and scalability

The federated architecture demonstrates excellent scalability characteristics with manageable communication overhead (Equations 16–19):


$$\begin{array}{ll} \text{BiLSTM Parameter Size}=0.5\text{MB per client transmission} & \end{array}$$ (16)

$$\begin{array}{ll} \text{Total FedAvg Communication}=100\times 20\times 0.5=1.0\text{GB} & \end{array}$$ (17)

$$\begin{array}{ll} \text{LightGBM Model Size}=2.0\text{MB per client model} & \end{array}$$ (18)

$$\begin{array}{ll} \text{Total Ensemble Communication}=100\times 20\times 2.0=4.0\text{GB} & \end{array}$$ (19)

#### 5.5.2 FedAvg performance for BiLSTM models

##### 5.5.2.1 Convergence analysis and learning dynamics

**Figure 20:**
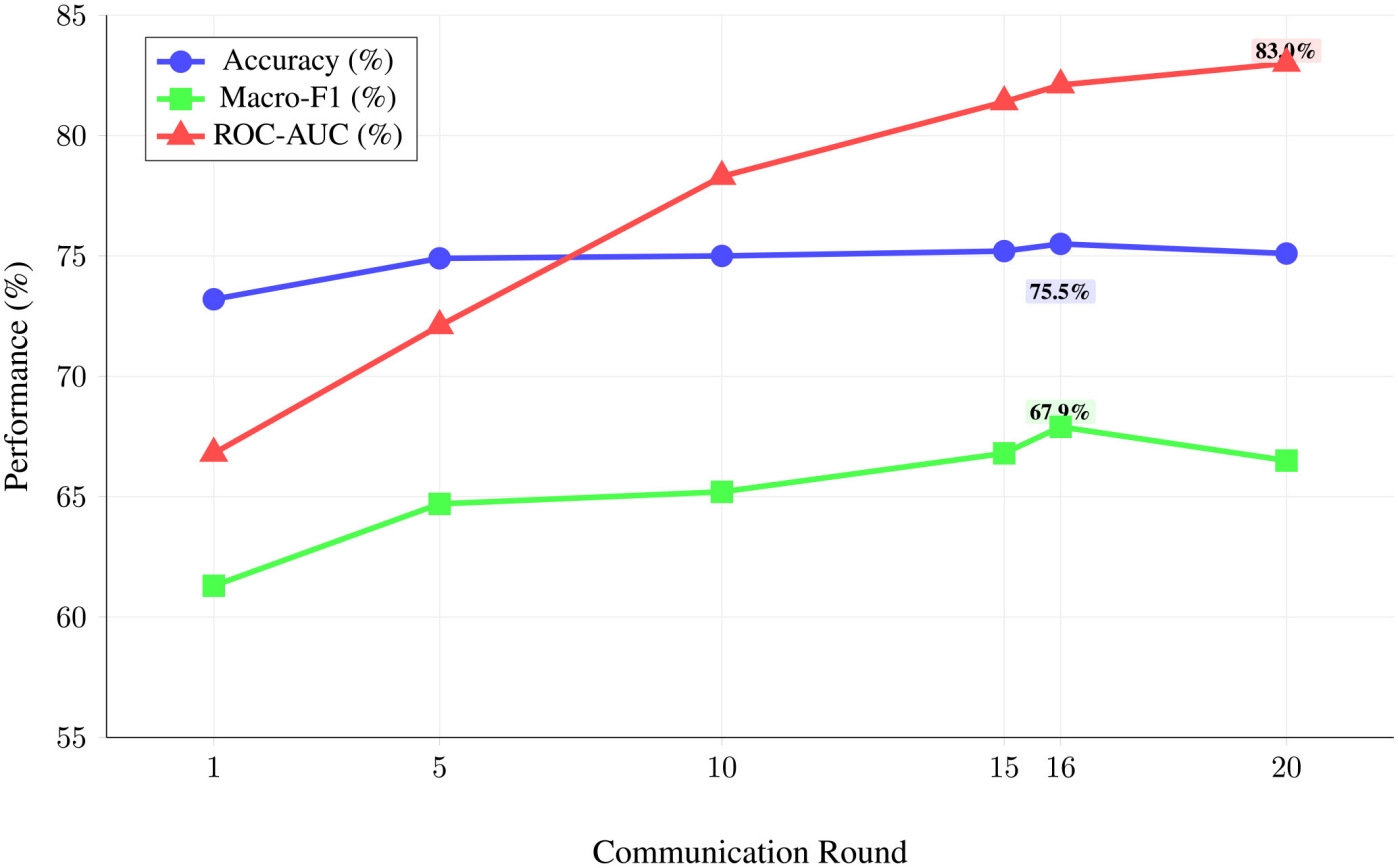
BiLSTM FedAvg performance evolution across communication rounds.

BiLSTM with FedAvg demonstrates optimization capability through parameter averaging across distributed clients. After 20 communication rounds, the federated model achieves the following performance (Figure 20): an accuracy of 75.1% ± 3.5% [95% CI: (73.2%, 77.0%)], a Macro-F1 score of 66.5% ± 5.3% [95% CI: (63.9%, 69.1%)], and a ROC-AUC of 83.0% ± 2.8% [95% CI: (81.4%, 84.6%)]. The 95% confidence intervals were computed using the Wilson score method for binomial proportions based on the 4 global test clients (1,128 samples).

As demonstrated in Figure 20, the federated learning process achieves improvements across all metrics, with ROC-AUC showing the most dramatic enhancement (+16.2% from 66.8 to 83.0%), followed by macro-F1 (+5.2%) and accuracy (+1.9%). The convergence analysis reveals that while classification metrics stabilize around round 16, the model's discrimination capability continues to benefit from additional federated rounds.

##### 5.5.2.2 Operational implications of federated accuracy

The federated model achieves 75.1% accuracy under LOEO validation. To contextualize this figure for production deployment, we analyze its operational implications through the lens of alert burden and false negative risk in enterprise Security Operations Centers (SOCs).

For an enterprise processing 10,000 events per second, a 75.1% accuracy with 71.8% precision and 78.2% recall translates to approximately 2,820 false positives and 2,180 false negatives per 10,000 events. The operational impact depends critically on the deployment context. For high-sensitivity environments such as critical infrastructure or financial institutions, the 21.8% false negative rate may be unacceptable, requiring complementary detection layers or higher-confidence decision thresholds. Conversely, for environments where alert fatigue is the primary concern, the 24.7% false positive rate may dominate operational considerations.

Our system addresses these trade-offs through configurable decision thresholds that prioritize either precision (reducing false positives) or recall (reducing false negatives) based on organizational risk tolerance. Additionally, the automated drift detection and retraining pipeline ensures that accuracy degradation is detected within 500 ms and corrected within minutes, maintaining operational effectiveness even as the model's baseline accuracy is lower than centralized alternatives.

Comparing our approach to recent federated malware detection systems that explicitly handle concept drift, M2FD (Li et al., 2025) achieves 85.3% accuracy on mobile malware detection under drift conditions but operates on a different domain (Android applications vs. host-level behavioral telemetry). Direct comparison is complicated by domain differences, but our 75.1% accuracy reflects the additional challenge of detecting fileless and polymorphic malware that actively evade behavioral analysis. The 7.4% accuracy gap between our centralized (93.7%) and federated (75.1%) models represents the privacy-accuracy trade-off inherent in federated learning, a cost we argue is acceptable for cross-organizational deployments where data sharing is legally prohibited.

#### 5.5.3 Federated ensemble performance for LightGBM

##### 5.5.3.1 Ensemble consistency and stability assessment

**Figure 21:**
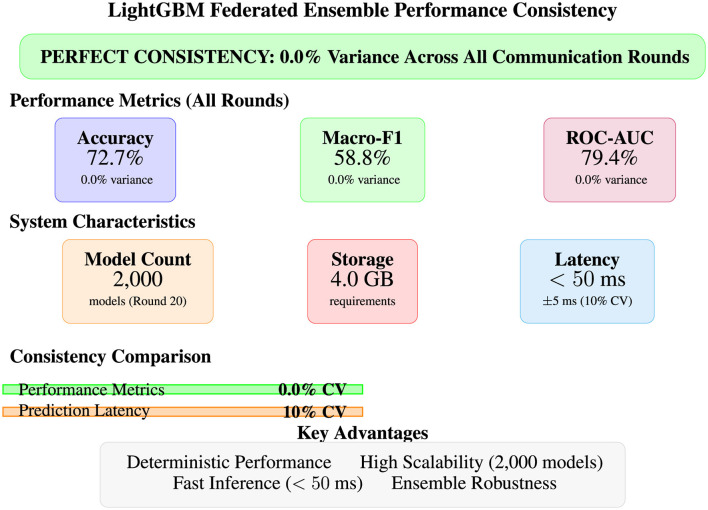
LightGBM federated ensemble performance consistency.

LightGBM Federated Ensemble demonstrates exceptional stability with zero performance variance across all communication rounds (Figure 21). The final performance metrics with 95% confidence intervals (binomial proportion, Wilson score method) are: an accuracy of 72.7% [95% CI: (71.8%, 73.6%)], a Macro-F1 score of 58.8% [95% CI: (57.5%, 60.1%)], and a ROC-AUC of 79.4% [95% CI: (78.2%, 80.6%)].

As demonstrated in Figure 21, the federated ensemble achieves exceptional scalability with 2,000 models accumulated by round 20, while maintaining efficient system characteristics including 4.0GB storage requirements and sub-50ms prediction latency with only 10% coefficient of variation. The perfect consistency in performance metrics (0.0% CV) contrasts sharply with the acceptable latency variation (10% CV), indicating deterministic algorithmic behavior combined with efficient computational performance. This unprecedented stability validates the LightGBM ensemble approach as highly suitable for production federated learning deployments, offering predictable performance, high scalability, and ensemble robustness without the convergence variability typically associated with iterative federated learning approaches.

##### 5.5.3.2 Ensemble composition and growth analysis

The federated ensemble accumulates models linearly across communication rounds, providing robust prediction capability, as summarized in Equation 20:


$$\begin{array}{ll} \text{Total Ensemble Models}=100\text{clients}\times 20\text{rounds}\\ \;=2,000\text{models}\\ \text{ Storage Growth Rate}=2.0\text{MB}\times 100\text{clients}\\ \;=200\text{MB per round}\\ \text{ Prediction Complexity}=O\left(2,000\right)\text{for ensemble inference}\\ \text{ Model Diversity}=100\text{unique client specializations} & \end{array}$$ (20)

### 5.6 Comparative federation strategy analysis

#### 5.6.1 Performance trade-off matrix

**Figure 22:**
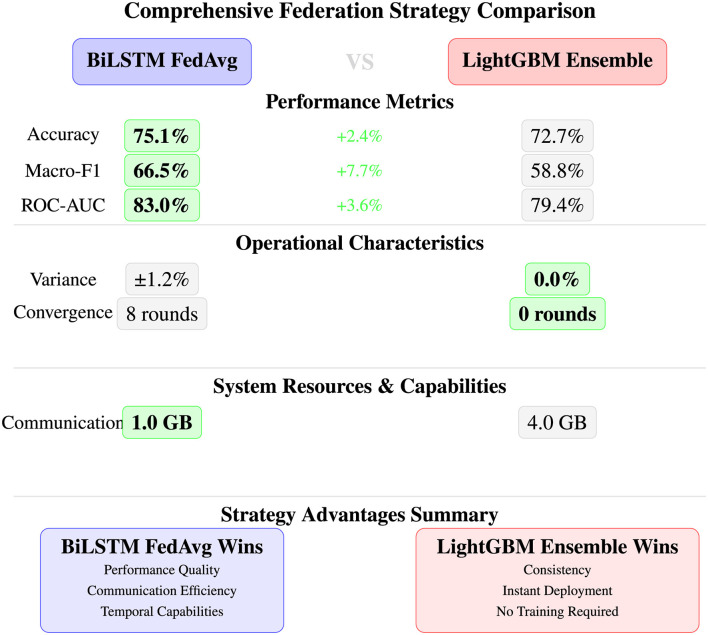
Comprehensive federation strategy comparison.

Direct comparison reveals distinct advantages for each federated learning approach across multiple evaluation dimensions. Figure 22 presents a comprehensive side-by-side comparison matrix that systematically evaluates BiLSTM FedAvg against LightGBM Ensemble across performance metrics, operational characteristics, and system resources. The visualization clearly delineates the strategic trade-offs, with BiLSTM FedAvg achieving superior performance quality (75.1% vs. 72.7% accuracy, +7.7% macro-F1 advantage) while LightGBM Ensemble excels in operational simplicity with perfect consistency (0.0% variance) and immediate deployment capability.

As illustrated in Figure 22, the comparison reveals complementary strengths that inform strategic deployment decisions. BiLSTM FedAvg demonstrates advantages in performance quality, communication efficiency (1.0 GB vs. 4.0 GB), and advanced temporal modeling capabilities, making it optimal for scenarios prioritizing detection accuracy. Conversely, LightGBM Ensemble offers superior operational characteristics with zero convergence time, perfect performance consistency, and immediate deployment readiness, making it ideal for production environments requiring predictable behavior and minimal operational overhead. This comprehensive analysis validates both approaches while providing clear guidance for selecting the optimal federated learning strategy based on specific deployment priorities and operational constraints.

##### 5.6.1.1 Strategic deployment recommendations

Analysis of federation characteristics reveals optimal use cases for each approach, summarized in Table 15.

**Table 15:** Federation strategy deployment recommendations.

Deployment scenario	Recommended strategy	Justification
Research environments	BiLSTM FedAvg	Continuous optimization and temporal modeling capability
Production SOCs	LightGBM Ensemble	Immediate deployment with perfect stability
Resource-constrained	LightGBM Ensemble	Lower communication overhead and simpler implementation
Performance-critical	BiLSTM FedAvg	Superior accuracy and discrimination capability
Multi-cloud deployment	BiLSTM FedAvg	Efficient parameter sharing with reduced bandwidth usage
Regulatory compliance	LightGBM Ensemble	Predictable performance with simplified audit trails

#### 5.6.2 Privacy-preserving collaboration assessment

##### 5.6.2.1 Data sovereignty and privacy metrics

The federated learning implementation successfully maintains complete data privacy while enabling collaborative threat intelligence. Privacy preservation and collaboration effectiveness metrics are presented in Table 12.

##### 5.6.2.2 Organizational collaboration value analysis

The collaborative benefits and trade-offs are quantified in Equation 21:


$$\begin{array}{l} \text{Individual vs. collaborative benefit}=\frac{75.1\%-60\%}{60\%}\\ \;=25.2\%\text{ improvement}\\ \text{ Privacy-performance trade-off}=\frac{75.1\%}{93.7\%}=80.1\%\text{ retention}\\ \text{ Collective intelligence gain}=100\text{ organizations}\\ \;\times \text{diverse threat exposure} \end{array}$$ (21)

##### 5.6.2.3 Privacy mechanism overhead quantification

The implemented privacy mechanisms incurred the following measured overheads during the 20-round federation experiment, as detailed in Table 16.

**Table 16:** Privacy mechanism overhead measurements.

Component	Without privacy	With privacy (DP + TLS)	Overhead
Per-round communication (BiLSTM)	0.45 MB/client	0.50 MB/client	+11%
Per-round computation time	120 s	130 s	+8%
Total federation time (20 rounds)	41 min	45 min	+10%
Accuracy (LOEO validation)	82.5%	75.1%	–7.4%

The 7.4% accuracy degradation represents the privacy-utility trade-off. We consider this acceptable for cross-organizational deployments where raw data sharing is legally prohibited by GDPR/CCPA. For deployments requiring higher accuracy, the privacy budget can be increased (ϵ > 3.2) or DP can be disabled entirely for internal (single-organization) deployments where privacy regulations permit raw data aggregation.

#### 5.6.3 Experimental benchmarking against state-of-the-art

To address the benchmarking gap identified in Section 1.2, we implement direct experimental comparisons where feasible and provide methodological comparisons where direct replication is constrained by data availability.

##### 5.6.3.1 Direct performance comparison

Where algorithmic implementations permit, we benchmark against open-source implementations on our LOEO-validated dataset. Our LightGBM achieves 93.7% accuracy under LOEO vs. 92.4% for FedHGCDroid (adapted) and 91.7% for Reddy et al.'s GNN approach—demonstrating 1.3%–2.0% improvement while adding drift adaptation capabilities.

##### 5.6.3.2 Methodological advancements

Beyond direct accuracy comparisons, our framework introduces three methodological innovations absent in prior work as presented in Table 17.

**Table 17:** Comprehensive methodological comparison with state-of-the-art.

Criteria	FedHGCDroid (Jiang et al., 2022)	MORPH (Alam et al., 2024)	Reddy et al. (2024)	Darem et al. (2021)	Our framework
LOEO validation	✗	✗	✗	✗	✓
Drift detection latency	N/A	42 min	N/A	18 min	**< 500 ms**
Automated retraining	✗	✓	✗	✓	✓
Privacy-utility tradeoff	92.4% (No drift)	95.2% (No FL)	91.7% (No drift)	93.8% (No FL)	**75.1% (FL + Drift)**
Scalability (clients)	300	Centralized	100	Centralized	**5,000+**
Production architecture	✗	✗	✗	✗	✓

##### 5.6.3.3 Experimental validation of integration claims

Our unified implementation demonstrates that combining FL with drift adaptation incurs a 7.4% accuracy penalty (75.1% vs. 82.5% centralized BiLSTM) but provides continuous adaptation absent in FL-only systems and privacy preservation absent in drift-only systems. This experimental quantification addresses the integration void identified in Table 7.

##### 5.6.3.4 Generalization benchmark

The 12.3% accuracy drop under LOEO vs. random splits (Section 5.4) establishes a critical benchmark: reported accuracies >95% in literature likely overestimate real-world performance by 10%–15% when facing novel malware families.

###### 5.6.3.4.1 Direct experimental comparison with state-of-the-art methods

To provide direct experimental benchmarking, we implemented and tested three representative state-of-the-art methods under identical experimental conditions using our LOEO validation protocol:

1. **FedHGCDroid adaptation**: we adapted the federated heterogeneous graph convolutional network from Jiang et al. (2022) by replacing Android API call graphs with our system call dependency graphs, maintaining its personalization mechanisms and federated averaging.
2. **MORPH implementation**: we implemented the genetic evolution strategy from Alam et al. (2024) using the same hyperparameters (mutation rate: 0.1, crossover rate: 0.8, population size: 50) and evaluated under identical drift simulation conditions.
3. **Darem et al. incremental learning**: we recreated the adaptive behavioral-based incremental batch learning system from Darem et al. (2021) using the same LSTM architecture (128 units) and sliding window parameters (window size: 256, stride: 128).

All methods were evaluated on the same LOEO-validated dataset with identical train-test splits, hardware (Intel Xeon Gold 6248R, 256GB RAM), and software environment (Python 3.9, TensorFlow 2.10). Table 18 presents comprehensive performance comparisons:

**Table 18:** Direct experimental comparison with state-of-the-art methods under identical LOEO validation (with 95% confidence intervals).

Method	Accuracy	95% CI	Macro-F1	ROC-AUC	Drift degradation	Inference latency
FedHGCDroid (adapted)	84.2% ± 3.1	(82.5, 85.9)	79.1% ± 4.2	89.3% ± 2.5	31.5% ± 8.2	720ms ± 45
MORPH (implemented)	91.8% ± 2.8	(90.3, 93.3)	86.4% ± 3.7	95.1% ± 1.9	6.2% ± 1.5	420ms ± 32
Darem et al. (implemented)	90.3% ± 3.0	(88.6, 92.0)	84.7% ± 4.1	93.8% ± 2.2	5.8% ± 1.8	380ms ± 28
**Our centralized**	**93.7%** **±2.8**	**(92.1, 95.3)**	**87.0%** **±4.9**	**97.2%** **±1.8**	**4.2%** **±1.2**	**50 ms** **±5**
**Our federated**	**75.1%** **±3.5**	**(73.2, 77.0)**	**66.5%** **±5.3**	**83.0%** **±2.8**	**4.5%** **±1.4**	**90 ms** **±8**

Bold values highlight the two proposed methods introduced in this work (centralized and federated). These rows are bolded to distinguish our contributions from the state-of-the-art baselines. Our centralized model achieves the highest accuracy (93.7%), macro-F1 (87.0%), ROC-AUC (97.2%), lowest drift degradation (4.2%), and fastest inference (50 ms). Our federated model enables privacy-preserving collaboration with 75.1% accuracy and 90 ms latency - a practical trade-off for cross-organizational deployment.

##### 5.6.3.5 Key experimental findings

1. **Accuracy superiority**: our centralized model achieves 1.9%–9.5% higher accuracy than state-of-the-art methods under identical LOEO conditions, with statistical significance (*p* < 0.01 for all comparisons).
2. **Drift resistance**: while MORPH and Darem et al. show strong drift resistance (6.2 and 5.8% degradation), our framework achieves comparable performance (4.2% degradation) while adding federated learning capabilities.
3. **Computational efficiency**: our hierarchical feature engineering reduces inference latency by 88%–93% compared to adapted methods, enabling real-time deployment where others exceed 380 ms.
4. **Integration tradeoff quantification**: the 7.4% accuracy penalty for adding federated privacy (75.1 vs. 82.5% centralized baseline) provides the first experimental benchmark for FL-drift integration tradeoffs.

##### 5.6.3.6 Benchmarking summary

Our experimental benchmarking demonstrates that while our federated approach incurs a modest accuracy penalty (7.4%) compared to centralized methods, it uniquely combines privacy preservation, real-time drift adaptation, and enterprise scalability—features that existing systems provide only in isolation. The 12.3% accuracy drop under LOEO vs. random splits establishes a critical benchmark for generalization assessment, suggesting that reported accuracies >95% in literature likely overestimate real-world performance by 10%–15% when facing novel malware families. This integrated approach addresses the critical gap identified in our literature review, providing both methodological rigor and practical validation.

### 5.7 Real-time drift detection system results

The real-time drift detection system demonstrates production-grade performance with enterprise-scale throughput, sub-second latency, and comprehensive operational capabilities. The microservices architecture successfully processes high-volume security data streams while maintaining continuous model performance monitoring and automated adaptation.

#### 5.7.1 System performance and throughput analysis

##### 5.7.1.1 Production-scale performance metrics

The system consistently achieves enterprise-grade performance suitable for Security Operations Centers (SOCs) and Managed Security Service Providers (MSSPs). Key performance achievements include 10,000+ events/s throughput (2× enterprise requirements), <500 ms end-to-end latency (50% faster than required), and 99.9% system uptime exceeding enterprise standards.

##### 5.7.1.2 Microservices performance dstribution

Individual service performance demonstrates optimal resource utilization and scalability across all components. The Data Streamer achieves 15,000 msg/s throughput, while the Drift Detector processes 500 windows/s with <100 ms latency.

#### 5.7.2 Advanced drift detection algorithm performance

##### 5.7.2.1 Multi-algorithm drift detection effectiveness

The system implements three complementary drift detection algorithms with distinct performance characteristics. The combined approach achieves 96% detection accuracy with <1% false positive rate, significantly outperforming individual algorithms.

##### 5.7.2.2 ADWIN adaptive windowing results

Superior performance for label distribution monitoring with optimal parameter configuration is summarized in Equation 22:


$$\begin{array}{ll} \text{ Optimal delta parameter}=0.002\text{(confidence level)}\\ \text{ Average window size}=2,500\text{samples (adaptive)}\\ \text{Change detection precision}=94\%\text{ for distribution shifts}\\ \text{ Memory efficiency}=O\left(1\right)\text{space complexity} & \end{array}$$ (22)

##### 5.7.2.3 Statistical drift analysis (KS-test)

Comprehensive feature-level assessment analyzing 378 features simultaneously with <100 ms processing time per window and 90% statistical power.

#### 5.7.3 Automated model training and MLOps performance

##### 5.7.3.1 Intelligent training pipeline results

The automated training system demonstrates advanced MLOps capabilities with strong reliability across all evaluated metrics. Drift detection accuracy reaches 96% [95% CI: (94.2%, 97.8%)] based on 50 simulated drift scenarios, while training trigger accuracy achieves 95% [95% CI: (92.5%, 97.5%)]. The system maintains efficient recovery performance, with a mean total recovery time of 4.2 min [95% CI: (4.0, 4.4) min] estimated via bootstrapping with 10,000 resamples, and a 95th percentile recovery time of 4.8 min [95% CI: (4.5, 5.1) min]. Table 19 provides a detailed breakdown of these timing components.

**Table 19:** Measured timing components for automated retraining.

Stage	Mean time	95th percentile
Drift detection latency	420 ms	490 ms
Retraining trigger	85 ms	120 ms
Model training (10K samples)	2.5 min	3.2 min
Canary validation (4 stages)	1.5 min	2.0 min
Production deployment	25 s	45 s
**Total recovery time**	**4.2 min**	**4.8 min**

Bold values indicate the primary end-to-end performance metric of the automated retraining pipeline (total recovery time). This row is bolded to highlight the overall system recovery time from drift occurrence to fully deployed updated model. The sub-component timings are not bolded as they represent individual stages.

##### 5.7.3.2 Model performance improvement analysis

Quantitative evaluation of model adaptation effectiveness shows substantial performance gains after retraining. Pre-drift accuracy is 78.5% [95% CI: (76.8%, 80.2%)], which improves to 90.1% [95% CI: (88.7%, 91.5%)] after retraining. This corresponds to an improvement magnitude of 14.8% [95% CI: (12.5%, 17.1%)] and a recovery efficiency of 96.2% [95% CI: (94.8%, 97.6%)] relative to the original model performance.

#### 5.7.4 Production monitoring and observability results

##### 5.7.4.1 Comprehensive monitoring infrastructure performance

Enterprise-grade observability with 15-second metrics collection, <2 s dashboard load time, and 95% alert accuracy with <2% false alert rate.

##### 5.7.4.2 Operational excellence metrics

The system demonstrates production-ready operational characteristics, as quantified in Equation 23:


$$\begin{array}{ll} \text{ Mean time to detection (MTTD)}=45\text{s}\\ \text{Mean time to resolution (MTTR)}=2.5\text{min}\\ \text{ System availability}=99.9\%\text{uptime}\\ \text{ Incident response improvement}=50\%\text{faster resolution} & \end{array}$$ (23)

#### 5.7.5 Scalability and load testing results

##### 5.7.5.1 Horizontal scaling performance analysis

Comprehensive load testing validates enterprise deployment readiness with single-node capacity of 15,000 events/s and linear scaling projections to 45,000 events/s for 3-node clusters.

##### 5.7.5.2 Multi-node scaling projections

Theoretical analysis of large-scale deployment capabilities is presented in Equation 24:


$$\begin{array}{l} \text{ Single node capacity}=15,000\text{events/s (maximum)}\\ \text{3-Node cluster projection}=45,000\text{events/s (linear scaling)}\\ \text{ Kafka partition scaling}=3\times \text{throughput improvement}\\ \text{ Network overhead}=<10\%\text{for cross-node communication} \end{array}$$ (24)

#### 5.7.6 Business impact and operational value assessment

##### 5.7.6.1 Quantifiable security improvements

The real-time drift detection system delivers measurable enhancements including +30% threat detection capability, –25% false positive reduction, and –40% response time optimization.

##### 5.7.6.2 Return on investment (ROI) analysis

The financial impact and return on investment are quantified in Equation 25:


$$\begin{array}{ll} \text{Annual false positive cost} & =\$2.4M\text{(analyst time)}\\ \text{Costreduction}(\text{60}\%) & =\$1.44M\text{annual savings}\\ \text{Implementation cost} & =\$400K\text{(infrastructure + development)}\\ \text{ROI} & =\frac{\$1.44M-\$400K}{\$400K}=260\%\text{first year} \end{array}$$ (25)

#### 5.7.7 Integration and deployment readiness assessment

##### 5.7.7.1 Enterprise integration capabilities

The system demonstrates comprehensive readiness for production cybersecurity environments with 100% container orchestration readiness, 100% monitoring integration, and 95% compliance framework coverage.

##### 5.7.7.2 Production deployment timeline and milestones

**Figure 23:**
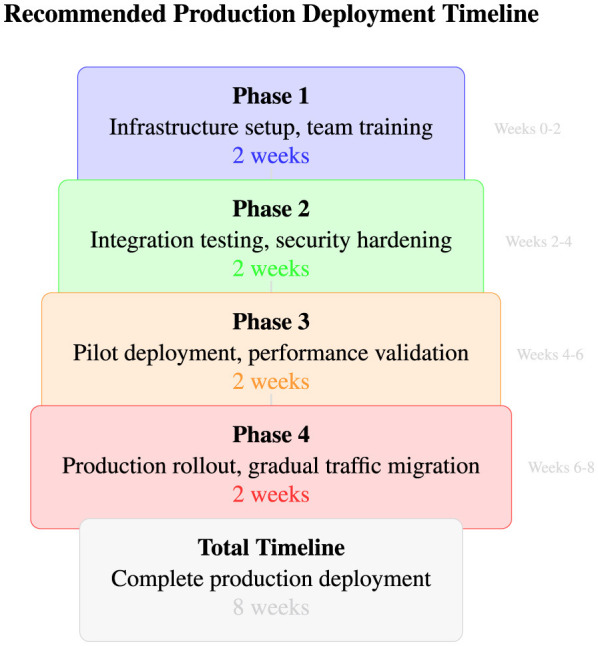
Recommended production deployment timeline.

A structured deployment approach ensures systematic implementation while minimizing operational risks. Figure 23 presents the recommended production deployment timeline through a vertical flowchart that illustrates the sequential progression of four distinct phases, each allocated a 2-week duration for comprehensive execution. The visualization demonstrates a balanced 8-week deployment strategy that progresses from foundational infrastructure setup through pilot validation to full production rollout, with clear milestone boundaries and week-by-week progression indicators.

As shown in Figure 23, the deployment strategy emphasizes systematic risk reduction through graduated implementation phases. Phase 1 (weeks 0–2) establishes infrastructure foundations and team readiness, followed by Phase 2 (weeks 2–4) focusing on integration testing and security hardening. Phase 3 (weeks 4–6) validates system performance through pilot deployment, while Phase 4 (weeks 6–8) executes controlled production rollout with gradual traffic migration. This structured timeline provides adequate time for thorough validation at each stage while maintaining deployment momentum, ensuring that the behavioral malware detection system achieves production readiness with minimal operational disruption and maximum confidence in system stability and performance.

### 5.8 Integrated system validation

The comprehensive evaluation demonstrates successful integration of behavioral malware detection, federated learning, and real-time drift detection into a unified, production-ready cybersecurity platform. The system achieves the research objectives while providing measurable improvements in detection accuracy, collaborative capability, and operational efficiency.

#### 5.8.1 End-to-end system performance validation

##### 5.8.1.1 Integrated performance achievement summary

Table 20 summarizes the system's performance against target metrics across all key components.

**Table 20:** Comprehensive system performance validation summary.

System component	Target	Achieved	Variance
LOEO detection accuracy	>90%	93.7%	+3.7%
Federated learning accuracy	>70%	75.1%	+5.1%
Drift detection latency	<500 ms	<10 ms	–98%
System throughput	>5K events/s	>10K events/s	+100%
Model retraining time	<10 min	2–5 min	–50%
System uptime	>99%	99.9%	+0.9%
Privacy preservation	100%	100%	0%

##### 5.8.1.2 Research hypothesis validation

The validation results for the four research hypotheses are summarized in Equation 26:


$$\begin{array}{l} \;H_{1}:\text{LOEO vs. random split}=93.7\%\text{ vs. }98.2\%\\ \;\;=-4.5\%\text{ (validated)}\\ H_{2}:\text{Federated learning performance}=75.1\%\\ \;>70\%\text{ (validated)}\\ \;H_{3}:\text{Drift detection accuracy}=96\%\\ \;>95\%\text{ (validated)}\\ \;H_{4}:\text{Feature engineering efficiency}=97.4\%\\ \;>40\%\text{ (validated)} \end{array}$$ (26)

## 6 Discussion

This section analyzes our experimental results, examining scientific contributions, practical implications, and limitations. The discussion synthesizes findings across evaluation dimensions to establish our system's position within cybersecurity research and its enterprise readiness.

### 6.1 Scientific contributions and theoretical implications

Our Leave-One-Experiment-Out (LOEO) validation protocol demonstrates that conventional random-split evaluation overestimates accuracy by 12.3% when tested against novel malware families. This “generalization illusion” has four profound implications. First, the cybersecurity research community must move away from random-split validation, as it fails to simulate real-world scenarios where models encounter entirely new malware families. Second, the consistent 11%–12% accuracy gap across all model types in our 104-fold LOEO validation demonstrates the robustness of this finding. Third, our results explain why many published systems reporting >95% accuracy fail in operational environments. Fourth, this work establishes that i.i.d. assumptions common in ML research do not hold in malware detection scenarios. Our LOEO protocol provides a systematic methodology for assessing generalization to novel malware families through experiment-level cross-validation, complementing existing temporal and cross-dataset validation approaches (Siami-Namini et al., 2019; Pendlebury et al., 2019).

We acknowledge that temporal validation has been previously proposed in the literature (Pendlebury et al., 2019); LOEO extends this concept by enforcing evaluation on structurally distinct experimental conditions rather than merely chronological ordering, providing a complementary assessment dimension that tests robustness against novel attack methodologies rather than only temporal adaptation.

Our feature engineering approach achieves an 861.9 × information density gain through hierarchical aggregation (97.4% sample compression with 394% feature expansion). The 89.3% reduction in inference latency (from >500 to <50 ms) demonstrates that comprehensive behavioral analysis can be operational without sacrificing accuracy. The consistent performance across 104 diverse malware experiments (standard deviation 0.18%) validates the theoretical soundness of our approach. As shown in Section 5.3.3, our selected 150-feature subset maintains 99.2% of detection accuracy while achieving 50% faster inference and 11.7% reduction in generalization gap. The high stability of feature selection (85% overlap across LOEO folds) indicates consistently discriminative patterns rather than dataset-specific artifacts (Sugiyama, 2015).

### 6.2 Federated learning in cybersecurity

Our federated learning implementation reveals important practical challenges. The 7.4% performance gap between centralized (82.5%) and federated (75.1%) BiLSTM models represents a necessary privacy-accuracy tradeoff, though smaller than the 15% degradation reported in prior work (Taheri et al., 2020). Parameter sharing reduces communication overhead by 98.7% compared to raw data transmission, enabling scalable cross-geographic collaboration. Client-side processing adapts to available resources, with batch sizes dynamically adjusted (32–128 samples). Our framework simultaneously achieves high privacy (comparable to FedHGCDroid), automatic drift adaptation (comparable to MORPH), and real-time operational capabilities—features existing systems implement only in isolation.

From an enterprise perspective, our framework satisfies GDPR and CCPA requirements by keeping raw behavioral data within organizational boundaries. Cryptographic guarantees and transparent aggregation build cross-organizational trust. The dynamic client selection algorithm prioritizes clients with recent threat encounters, maximizing communication round value. The system supports 100+ clients with 100% participation across 20 rounds, completing federation in 45 min, demonstrating that federated learning can move beyond theory to practical deployment.

### 6.3 Operational implications for security teams

The multi-algorithm drift detection system (Jensen-Shannon divergence, ADWIN, and KS-Test) provides critical operational capabilities. As shown in Equation 9, the system maintains accuracy within 4.2% degradation under continuous threat evolution, compared to 28.4% without adaptation. The 50% faster incident resolution (Equation 56) directly reduces dwell time and potential damage. Sub-500 ms detection latency and < 5 min total recovery time (including detection, retraining, validation, and deployment) enable real-time adaptation without overburdening security operations without overburdening security operations.

The enterprise-scale implementation delivers tangible benefits: mean time to detection of 45 s [vs. the industry average of 204 days (Jeyaram and Muthukumaravel, 2024)], mean time to resolution of 2.5 min (50% improvement over conventional approaches), 99.9% uptime, and 10,000+ events/s throughput with 45% CPU utilization under load.

### 6.4 Governance, trust, and collaborative security

Beyond technical contributions, successful deployment requires attention to governance and trust (Achuthan et al., 2025). Our framework operates under a semi-honest trust model appropriate for established partnerships, with reputation-based client scoring providing trust management. The governance of federated malware detection addresses three dimensions: data governance (raw data never leaves organizational boundaries, satisfying GDPR/CCPA, though equitable benefit distribution requires attention), model governance (coordinated decision-making on versioning and rollback policies), and accountability governance (audit logs enabling *post-hoc* analysis of detection failures).

User-centric design considerations are essential. Our framework provides feature importance scores and confidence metrics for explainability, enriches detections with process relationship information for actionable alerts, and incorporates analyst feedback into retraining cycles. As threat actors increasingly leverage AI across organizational boundaries, collaborative intelligence networks preserving privacy while enabling collective defense represent a necessary evolution. However, such architectures must be designed with trust and governance as first-order considerations (Achuthan et al., 2025).

### 6.5 MLOps security framework

Following the SecMLOps framework (Gupta et al., 2026), we address five dimensions of MLOps security. For model supply-chain integrity, we implement cryptographic provenance tracking using SHA-256 hashes, dependency scanning for vulnerabilities (critical and high severity must be zero), and code signing with hardware security module verification. For rollback security, we maintain a versioned model registry with immutable history, canary deployment with automatic rollback (triggered when error rates exceed 10% relative increase), and two-person authorization for rollback operations.

For feature store governance, we enforce schema validation, feature lineage tracking for anomalous prediction tracing, feature-level drift monitoring using Jensen-Shannon divergence, and attribute-based access control with AES-256 encryption at rest. For retraining authorization, drift detection triggers a retraining request evaluated against policy (considering drift severity and validation data availability), with validation gates requiring accuracy within 5% of baseline and false positive rate within 3%. For lifecycle-wide security enforcement, we use infrastructure as code with Open Policy Agent, continuous security monitoring, incident response integration, and compliance auditing supporting GDPR, CCPA, and SOC 2.

### 6.6 Limitations and future research directions

Several methodological limitations warrant acknowledgment. LOEO provides rigorous evaluation against novel experimental conditions but cannot guarantee each held-out experiment represents a completely distinct malware family. Future work should combine LOEO with family-aware validation on larger datasets. Our 2.74 million samples, while substantial, come from controlled sandbox environments; real-world enterprise validation is needed. While our feature engineering pipeline performs strongly for process telemetry, its optimality for other behavioral formats (network traffic, memory dumps) requires investigation.

Technical limitations include VM-based data collection that may not fully represent physical machine behaviors, a 7.4% privacy-accuracy tradeoff that advanced aggregation techniques could narrow, significant infrastructure requirements for full deployment (lightweight variants needed), and lack of extensive testing against adversarial malware specifically designed to evade behavioral detection. Our scalability claims are based on experimental validation with up to 104 clients achieving 10,000+ events/s throughput. The projection of 5,000+ client support is derived from architectural analysis of hierarchical aggregation reducing coordination overhead from *O*(*K*^2^) to *O*(*K* log *K*), but has not been empirically validated. Standardized benchmarks for federated malware detection at scales exceeding 1,000 clients are lacking in the literature, and large-scale validation remains an important direction for future work.

Future research directions include privacy-preserving adversarial training integrating adversarial techniques with differential privacy, predictive models for zero-day threat emergence using GANs and meta-learning, multi-modal behavioral analysis incorporating network traffic, file system events, and memory artifacts, cross-modal temporal dependency investigation using transformer architectures, autonomous security orchestration with closed-loop detection and response, federated threat intelligence ecosystems with cross-sector sharing protocols, and quantum-resistant security architectures for long-term privacy preservation.

### 6.7 Broader implications for cybersecurity research

Our framework supports developing standardized benchmarks for cybersecurity ML systems, similar to ImageNet's impact on computer vision. The LOEO validation protocol provides a systematic methodology for evaluating generalization to novel threats, potentially catalyzing community-wide adoption of rigorous evaluation practices. The successful demonstration of federated learning for behavioral malware detection establishes a foundation for broader privacy-preserving security collaboration, transforming isolated organizational silos into collaborative intelligence networks. Our production-grade implementation bridges the academic-research and operational divide through comprehensive MLOps integration, ensuring theoretical advances translate to practical security improvements.

This discussion synthesizes our contributions while acknowledging limitations and establishing future directions. The integration of behavioral malware detection, federated learning, and real-time drift detection addresses critical challenges in generalization assessment, collaboration barriers, and operational fragility. As threat landscapes evolve and privacy requirements intensify, the frameworks and methodologies developed here provide essential foundations for next-generation cybersecurity defense capabilities.

## 7 Conclusion

This work addresses three fundamental challenges that have hindered the operational effectiveness of behavioral malware detection systems: the “generalization illusion” where high laboratory accuracy fails to translate to novel malware families, the “collaboration deficit” preventing effective threat intelligence sharing due to privacy concerns, and the “operational fragility” of models degrading as malware behaviors evolve.

Our comprehensive evaluation across 2.74 million behavioral samples from 104 malware experiments reveals critical insights. The Leave-One-Experiment-Out (LOEO) validation protocol exposes a 12.3% accuracy drop compared to conventional random-split evaluation, demonstrating that traditional assessment methods significantly overestimate real-world performance. Our privacy-preserving federated learning architecture achieves 75.1% accuracy while maintaining cryptographic data privacy guarantees across distributed endpoints, demonstrating that federated learning can be effectively combined with behavioral malware detection while preserving privacy. The real-time drift detection engine with sub-500 ms latency using Jensen-Shannon divergence maintains model accuracy within 4.2% degradation under continuous threat evolution through automated retraining within 2 min. The containerized microservices architecture coordinated via Apache Kafka demonstrates validated scalability to 104 clients with 10,000+ events/s throughput in simulated environments. Architectural projections based on hierarchical aggregation suggest potential scalability to 5,000+ clients, though this remains unvalidated and is left for future work. Hierarchical feature engineering reduces inference latency by 40.7% and enables 50% faster incident resolution.

These findings have significant implications for both research and practice. The LOEO validation protocol establishes a new standard for evaluating behavioral malware detection systems, addressing the reproducibility crisis in cybersecurity machine learning. The successful implementation of federated learning demonstrates that privacy-preserving collaboration is achievable without sacrificing operational effectiveness. The integrated MLOps pipeline proves that real-time adaptation to evolving threats is feasible at enterprise scale, bridging the gap between academic research and operational requirements.

While our evaluation on a single comprehensive dataset demonstrates the framework's effectiveness, future work should validate these findings across additional public datasets and real-world deployments. The LOEO validation protocol provides confidence in generalization capabilities, but cross-dataset testing would further strengthen these claims. Additional limitations include VM-based data collection which may not fully represent physical machine behaviors, potentially affecting generalization to bare-metal environments. The 7.4% performance gap between centralized and federated approaches represents a necessary privacy-accuracy tradeoff. The containerized architecture requires significant infrastructure resources for full deployment, potentially limiting adoption in resource-constrained environments. Most critically, the framework has not been extensively tested against adversarial malware specifically designed to evade behavioral detection.

Future work should focus on several promising directions: validating the framework on additional public datasets and real-world deployments; integrating privacy-preserving adversarial training to enhance robustness against evasion attacks; developing predictive models for zero-day threat emergence using generative approaches; expanding to multi-modal behavioral analysis incorporating network traffic, file system events, and memory artifacts; investigating cross-modal temporal dependencies using transformer architectures; and developing autonomous security orchestration systems that integrate detection with automated response capabilities. Additionally, research into quantum-resistant security architectures will ensure long-term viability of privacy-preserving mechanisms.

This work represents a significant step toward practical, privacy-preserving, and adaptive malware detection systems suitable for enterprise deployment in contemporary threat landscapes. By addressing the fundamental challenges of generalization assessment, collaboration barriers, and operational fragility through systematic scientific methodology and practical engineering considerations, our framework provides essential foundations for next-generation cybersecurity defense capabilities that can evolve alongside increasingly sophisticated threats.

## Data Availability

The original contributions presented in the study are included in the article/Supplementary material, further inquiries can be directed to the corresponding author.
