# Supplementary material for: Advanced behavioral malware detection: a comprehensive MLOps framework with federated learning and real-time drift detection
Source: Front Artif Intell. 2026 May 11;9:1811692. doi: 10.3389/frai.2026.1811692 (PMC13199286; doi:10.3389/frai.2026.1811692)
Supplement: Supplementary file 1 [file Data_Sheet_1.pdf]

supp

m.elhajj

April 2026

Reproducibility and Data Availability Statement

To address the reproducibility crisis in cybersecurity machine learning and to enable independent verification of our results, we provide a comprehensive reproducibility package.

Code Release

The complete source code for this work is released under the MIT license and includes:

- Feature engineering pipeline (hierarchical aggregation, rolling window statistics, process relationship clustering)
- LOEO cross-validation framework with experiment-to-fold mapping
- Federated learning implementation (FedAvg for BiLSTM and Federated Ensemble for LightGBM)
- Real-time drift detection engine (JSD, ADWIN, KS-Test with ensemble voting)
- Automated retraining pipeline with canary deployment
- Evaluation scripts for all metrics reported in this paper

Configuration Files and Containerization

We provide the following to ensure environment reproducibility:

- Docker Compose manifests for the complete MLOps stack (Kafka, Zookeeper, Prometheus, Grafana, and detection services)
- Dockerfiles for each service with pinned base images and dependency versions
- Configuration files for hyperparameters, feature selection thresholds, and drift detection parameters

- requirements.txt with exact Python package versions (pip freeze output)
- conda environment.yml for Anaconda-based reproduction

Data Sharing

Due to legal restrictions on malware distribution, the full dataset cannot be publicly released. However, we provide:

- **Synthetic anonymized subset**: A representative subset (10% of the data, 274,014 samples) with preserved statistical properties (means, variances, correlations) and class distribution (55.3% benign, 44.7% malicious). Generated using a Gaussian copula model fitted on the original data.
- **Data collection code**: Scripts to reproduce the data collection pipeline using public malware sources (VirusShare, SOREL-20M) and benign workload automation (AutoHotkey scripts).
- **Feature-extracted dataset**: Pre-computed feature vectors (378 dimensions) for all 2.74M samples, which can be shared without raw process telemetry.
- **Experiment metadata**: CSV file mapping each of the 104 experiments to its malware family, execution variant, system configuration, and network condition.

LOEO Fold Reproduction

To enable exact reproduction of our 104-fold LOEO validation:

- We provide `loeo_folds.csv` containing the exact experiment-to-fold mapping (104 rows, each specifying which experiment is held out as test)
- Python code (`reproduce_loeo_folds.py`) to regenerate the splits using the same deterministic algorithm
- Verification script that confirms the generated folds match our reported results (accuracy difference ¡0.1%)

Hardware and Software Specifications

All experiments were conducted on the following environment:

Table 1: Hardware and software specifications for reproducibility

Component	Specification
CPU	Intel Xeon Gold 6248R @ 3.0GHz (20 cores, 40 threads)
RAM	256GB DDR4 @ 2933MHz
Network	10GbE (10,000 Mbps)
Storage	2TB NVMe SSD (Samsung PM983)
OS	Ubuntu 20.04.6 LTS (Focal Fossa)
Docker	20.10.23
Python	3.9.16
TensorFlow	2.10.1
LightGBM	3.3.5
scikit-learn	1.2.2
NumPy	1.23.5
Pandas	1.5.3
Apache Kafka	3.0.0 (with Zookeeper 3.8.0)
Prometheus	2.42.0
Grafana	9.3.2

Random Seeds

To ensure deterministic reproducibility, all random seeds are fixed:

- Python random: `random.seed(42)`
- NumPy: `np.random.seed(42)`
- TensorFlow: `tf.random.set_seed(42)`
- PyTorch (if used): `torch.manual_seed(42)`
- scikit-learn: `random_state=42` in all functions
- LightGBM: `random_state=42`

The seed initialization code is provided in `set_random_seeds.py`.

Hyperparameter Search Budget and Protocol

We conducted hyperparameter optimization using the following protocol:

Search Budget:

- Total compute time: 720 CPU-hours (30 days on 1 CPU core)
- Total GPU time (for BiLSTM): 240 GPU-hours (10 days on 1 NVIDIA A100)
- Number of evaluations: Random Forest (100), LightGBM (150), BiLSTM (50)

Random Forest Hyperparameter Search:

- Method: Grid search with 5-fold cross-validation
- Search space: n_estimators [100, 200, 500]; max_depth [None, 10, 20, 30]; min_samples_split [2, 5, 10]
- Total combinations: $3 \times 4 \times 3 = 36$ grid points (expanded to 100 with random sampling)
- Optimal configuration: n_estimators=200, max_depth=None, min_samples_split=2

LightGBM Hyperparameter Search:

- Method: Bayesian optimization (Tree-structured Parzen Estimator) with 5-fold cross-validation
- Search space: learning_rate [0.01, 0.3] (log uniform), num_leaves [20, 100] (integer), max_depth [3, 15] (integer), lambda_l1 [1e-5, 1e-1] (log uniform), lambda_l2 [1e-5, 1e-1] (log uniform)
- Total evaluations: 150 (50 initial random, 100 Bayesian iterations)
- Optimal configuration: learning_rate=0.1, num_leaves=50, max_depth=8, lambda_l1=0.01, lambda_l2=0.1

BiLSTM Hyperparameter Search:

- Method: Grid search with early stopping (patience=5)
- Search space: layers [1, 2, 3]; units [64, 128, 256]; dropout [0.3, 0.5, 0.7]; learning_rate [1e-4, 1e-3, 1e-2]
- Total combinations: $3 \times 3 \times 3 \times 3 = 81$ evaluations
- Optimal configuration: layers=2, units=128, dropout=0.5, learning_rate=1e-3
- Early stopping patience: 5 epochs, restoring best weights

Reproducing Hyperparameter Search: The exact search space definitions, cross-validation splits, and evaluation metrics are provided in `hyperparameter_search/` directory, including:

- `rf_grid_search.py` - Random Forest grid search with 5-fold CV
- `lgbm_bayesian_search.py` - LightGBM Bayesian optimization
- `bilstm_grid_search.py` - BiLSTM grid search with early stopping
- `search_results.csv` - All evaluated configurations and their performance

Contact for Data Access

For access to the full feature-extracted dataset (378-dimensional feature vectors for all 2.74M samples) or the raw process telemetry under data use agreements, please contact the corresponding author at mhajj@aou.edu.lb. Requests from academic researchers will be processed within 14 days.
